# How mHealth can facilitate collaboration in diabetes care: qualitative analysis of co-design workshops

**DOI:** 10.1186/s12913-020-05955-3

**Published:** 2020-11-30

**Authors:** Meghan Bradway, Rebecca L. Morris, Alain Giordanengo, Eirik Årsand

**Affiliations:** 1 Norwegian Centre for E-health Research, University Hospital of North Norway, P.O. Box 35, N-9038 Tromsø, Norway; 2 Department of Clinical Medicine, Faculty of Health Sciences, UiT The Arctic University of Norway, 9037 Tromsø, Norway; 3 NIHR Greater Manchester Patient Safety Translational Research Centre, Centre for Primary Care, The University of Manchester, Oxford Rd, Manchester, M13 9PL UK; 4 Department of Computer Science, Faculty of Science and Technology, UiT The Arctic University of Norway, 9037 Tromsø, Norway

**Keywords:** Patient-gathered data, Data-sharing, Co-design, mHealth, App, Health care providers

## Abstract

**Background:**

Individuals with diabetes are using mobile health (mHealth) to track their self-management. However, individuals can understand even more about their diabetes by sharing these patient-gathered data (PGD) with health professionals. We conducted experience-based co-design (EBCD) workshops, with the aim of gathering end-users’ needs and expectations for a PGD-sharing system.

**Methods:**

*N* = 15 participants provided feedback about their experiences and needs in diabetes care and expectations for sharing PGD. The first workshop (2017) included patients with Type 2 Diabetes (T2D) (*n* = 4) and general practitioners (GPs) (*n* = 3). The second workshop (2018) included patients with Type 1 Diabetes (T1D) (*n* = 5), diabetes specialists (*n* = 2) and a nurse. The workshops involved two sessions: separate morning sessions for patients and healthcare providers (HCPs), and afternoon session for all participants. Discussion guides included questions about end-users’ perceptions of mHealth and expectations for a data-sharing system. Activities included brainstorming and designing paper-prototypes. Workshops were audio recorded, transcribed and translated from Norwegian to English. An abductive approach to thematic analysis was taken.

**Results:**

Emergent themes were mHealth technologies’ impacts on end-users, and functionalities of a data-sharing system. Within these themes, similarities and differences between those with T1D and T2D, and between HCPs, were revealed. Patients and providers agreed that HCPs could use PGD to provide more concrete self-management recommendations. Participants’ paper-prototypes revealed which data types should be gathered and displayed during consultations, and how this could facilitate shared-decision making.

**Conclusion:**

The diverse and differentiated results suggests the need for flexible and tailorable systems that allow patients and providers to review summaries, with the option to explore details, and identify an individual’s challenges, together. Participants’ feedback revealed that both patients and HCPs acknowledge that for mHealth integration to be successful, not only must the technology be validated but feasible changes throughout the healthcare education and practice must be addressed. Only then can both sides be adequately prepared for mHealth data-sharing in diabetes consultations. Subsequently, the design and performance of the joint workshop sessions demonstrated that involving both participant groups together led to efficient and concrete discussions about realistic solutions and limitations of sharing mHealth data in consultations.

**Supplementary Information:**

The online version contains supplementary material available at 10.1186/s12913-020-05955-3.

## Background

As a medical society, we have increased our knowledge about diabetes beyond managing the cornerstones of self-management: blood glucose, physical activity, medication and diet. We have recently unmasked the effects of less well-known factors as sleep, stress or even temperature, on blood glucose levels [1]. While it is theoretically ideal to understand all factors that affect a disease, in order to effectively treat it, it also inadvertently puts added pressure on healthcare providers (HCPs) and patients to not only track these factors but also understand and react to them. In fact, it was only 50 years ago, with the invention of the first commercial glucose meter, that patients were given the ability to check their blood glucose at home [2]. Since then, medical devices for diabetes have been developed alongside the necessary systemic changes to the medical system that are required to effectively use such new technologies. However, this trend has shifted as commercial technology, such as mobile health (mHealth) apps and devices, now offers patients the ability to easily track all of the indicated disease factors that are expected of them, often without oversight from medical professionals [3].

Lately, the use of mHealth technologies has become common practice for diabetes self-management [4]. For example, by connecting one’s smartphone app to a blood glucose meter and wearable activity tracker, one can automatically combine blood glucose levels with how physically active they are as well as manually entered food and medication intake. Such measures are considered patient-gathered data (PGD) and allow a user to track how their self-management activities affect their health outcomes [5]. With this stored history, the next time an individual chooses to undergo a similar combination of activities, they could easily identify, for example, how they chose to eat or what dose of insulin was effective or not for that situation. However, this information is only effective if used correctly; not everyone is able to process and make connections for all of this information on their own. Therefore, while mHealth provides clear potential benefits, there is only so much most individuals can understand without the complementary medical knowledge of the disease itself. This is where the potential of sharing one’s own data from their mHealth tools with HCPs can benefit both the patient’s understanding of their own health and the provider’s understanding of how to best practice personalized and evidence-based medicine.

Unfortunately, when it comes to introducing mHealth and PGD in the clinic, both parties have differing ideas as well as concerns and unanswered questions. Providers have noted concerns about data overload and how to relate to the data for clinical decision-making [6]. Patients are concerned with how providers can effectively use this information to give personalized health recommendations [7]. Despite a growing effort to research these technologies, most research focuses on exploring the topics of technical possibilities, feasibility, usability and policy issues [8], with little focus on how both patients and providers can use PGD together. This is not only due to the concerns and questions mentioned above but also because the gap in disease knowledge between patients and providers has traditionally been too great [9].

This gap has lately been shrinking thanks to mHealth, which adds a new dimension of diabetes management – enables greater self-efficacy, disease understanding, especially among technology savvy people. In fact, in the field of mHealth, patients’ have become vastly more knowledgeable, and are even considered “experts” by some [10]. By gaining insight into their own disease self-management, patients are now more capable of bringing this understanding and PDG to consultation discussions with their healthcare providers [11, 12]. Therefore, there is a need for data-sharing systems to be able to transfer, structure and present this data in a way that facilitates collaborative discussions and shared decision-making in diabetes care. Previous studies in the field of health technology have provided knowledge regarding the needs of data integration and patients’ and HCPs’ expectations and their needs from data-sharing technologies. The majority of these studies have gathered information from patients [13] and providers [14] separately. However, other studies also show that when both end-user groups were engaged together in development discussions, more concrete and realistic solutions can be identified [15].

Experience-based co-design (EBCD) (hereby referred to as co-design) allows patients, and providers to impose their collaborative insights on the design and development of the tools and services that they are eventually meant to use [16]. “Happenings become experiences when they are digested, when they are reflected on, related to general patterns and synthesised” [17]. This describes the general use scenario of those who use mHealth technologies for chronic illness self-management; recording, reviewing or reflecting and synthesizing an understanding of their health experiences. Unfortunately, many “patient-centred” research efforts do not always involve patients or other end-users in such design, and/or development [18, 19]. By considering patients as “experts” in their own self-management and providers as, of course, experts in the disease mechanics, we acknowledge that both parties can bring complementary knowledge and skills to diabetes care. Ideally, this is considered the process of shared decision-making, which is characterized by providers and patients collaborating to make decisions about the patient’s health, with a balanced focus on both hard clinical evidence as well as the patient’s priorities and values [20]. This suggests the necessity of engaging both main end-users in co-design to design and develop the technology that they will use, together [21].

In this paper, we present the qualitative analysis of transcripts and paper-prototypes from two co-design workshops involving both patients and HCPs regarding the design of a system to share patient-gathered self-management data during diabetes consultations. These workshops were conducted as part of a larger research project to create and test a system for sharing PGD between patients and providers, called the “Full Flow of Data Between Patients and Healthcare Services” project (2016–2020) [22]. Previous workshops within the same research project reported the differences in self-management foci and challenges between those with T1D and T2D, as well as differences in how specialists and GPs meet their patients and their clinical practice needs. These results were published elsewhere [23]. In this paper, we build upon this knowledge, and the input from co-design, to design a system for sharing PGD during diabetes consultations. We focus on our end-users’ intentions for the use of, needed functionalities, ideal discussion and collaboration that can and should be generated from sharing PGD.

## Objective

By arranging two co-design workshops, where patients and HCP together discuss expectations and design ideas for an mHealth data-sharing system for diabetes, we aim to understand how a system can present patient-gathered mHealth data and be used effectively by both parties to facilitate shared-decision making and collaboration in diabetes care.

## Methods

Two co-design workshops (*N* = 15) were conducted with the aim of inviting both stakeholder groups to discuss the concept of sharing and using patient-gathered self-management data during diabetes consultations. The first involved patients with type 2 diabetes (T2D) (*n* = 4) and GPs (*n* = 3) (2017) and the second involved patients with type 1 diabetes (T1D) (*n* = 5), diabetes specialists (*n* = 2) and a nurse (2018). The workshops were held in Norwegian, the participants’ native language.

### Recruitment

Participants were invited to attend the workshops at the Siva Innovation Centre in Tromsø, Norway. Convenience sampling was used to expedite recruitment and draw from a population with experience or interest in the particular field of mHealth for diabetes self-management. Patients were recruited by messages sent through the Diabetes Diary app [24], which is available on Google Play app store. At the time of recruitment, there were approximately 7000 downloads of this app in Norway. Patient participants had to be 18+ years with either T1D or T2D and be willing to travel to Tromsø, Norway for the workshop. All who expressed interest and met inclusion criteria were invited to participate. All participants presented a signed consent form prior to the workshop. HCPs, who currently see patients with diabetes, were recruited via e-mail requests. Participants were given the option to withdraw their participation at any time.

### Discussion guides and workshop activities

During each daylong workshop, patients and clinicians were split into their respective groups in the morning. Following a common lunch, all participants took part in a joint session in the afternoon. The intention of joining both groups was to allow participants to present their views to each other and to discuss and correct assumptions and expectations regarding mHealth technologies and data-sharing during consultations. A moderator used a semi-structured discussion guide, which was developed by the co-authors (see Additional file 1).

Two story-boards, describing T1D care and T2D care, were split into three main sections illustrating the following: experiences and topics surrounding patients’ own self-management, the healthcare providers’ clinical practice and experiences, and the consultation between both patients and providers, which was used only during the joint session. In both of the separate patient and provider sessions, participants filled out post-it notes in response to questions, presented them orally to the group and then placed the notes on the story-board that corresponded to each of the three situations. This allowed them to form their own opinions before engaging in group discussions. During the joint session, participants were asked to create, and then describe how to use, his or her own paper-prototype of an ideal data-sharing system. Paper cut-outs that represented functionalities and features of the system’s interface were provided. These included cartoon representations of data sources, such as mobile phones, wearables and sensors, data types, such as blood glucose and physical activity, how to display data, such as graphs, arrows and scales, and computer screen, through which the system is meant to be accessed.

### Thematic analysis

After each workshop, single-page summaries were made by the research team, within a month following each co-design workshop, and sent to all participants. Participants were encouraged to correct these reports, comment or ask any additional questions before further analysis was performed.

All sessions were audio recorded, transcribed and translated into English by a native Norwegian speaker, and de-identified. As not all in the research team were present during all sessions, before more detailed analysis took place, narrative summaries for each of the six co-design sessions were created. Co-authors discussed the summaries to ensure collective understanding of the transcripts, e.g. what was produced that was directly related to the research questions and what unexpected yet relevant additional information was provided. To identify patterns within and across the participants’ feedback while also addressing the research questions, a thematic analysis was used. As it is difficult to separate one’s self from their research experiences and background knowledge, this thematic analysis included iterative use of deductive and inductive reasoning to structure and report the transcripts, i.e. an abductive approach [25]. The deductive approach first generated themes, based upon discussion guide questions that participants responded to, from a small selection of the transcript, which are described as “analytic inputs” by Braun et al. [26, 27]. These themes then direct the combination of emergent salient concepts, i.e. the inductive approach; while emergent concepts were identified and grouped as primary and secondary codes, relevant codes were selected and combined into sub-themes and assigned, based upon reasonable association, to agreed-upon themes [28]. An example of this process is provided in Table 1. Quotations will be formatted with brackets indicating omitted words, e.g. “it”, “they”, that are replaced with the words to which these articles refer.

**Table 1:** Abductive approach to analysis process of categorizing quotable text from the transcript into codes, followed by the grouping of codes into progressively higher-level themes

Deductive Analysis →		←Inductive analysis			
Narrative summary for joint T1D session	Example of agreed-upon theme	Codes grouped under concept/ Sub-theme	Secondary codes	Initial codes	Example from transcript
• Research questions asked • Impressions of major topics and concepts presented in the transcripts by both patients and providers	Data-sharing system	Concerns	• Which data to share/look at • Time capacity of consultations	• Question: how much data can incorporate into consultation? • Preference to see selected/relevant data	“Could you possibly assimilate so much data?...How much data can you incorporate into a [15-min] consultation?” (Specialist2)

## Results

### Demographics

**Fig. 1:**
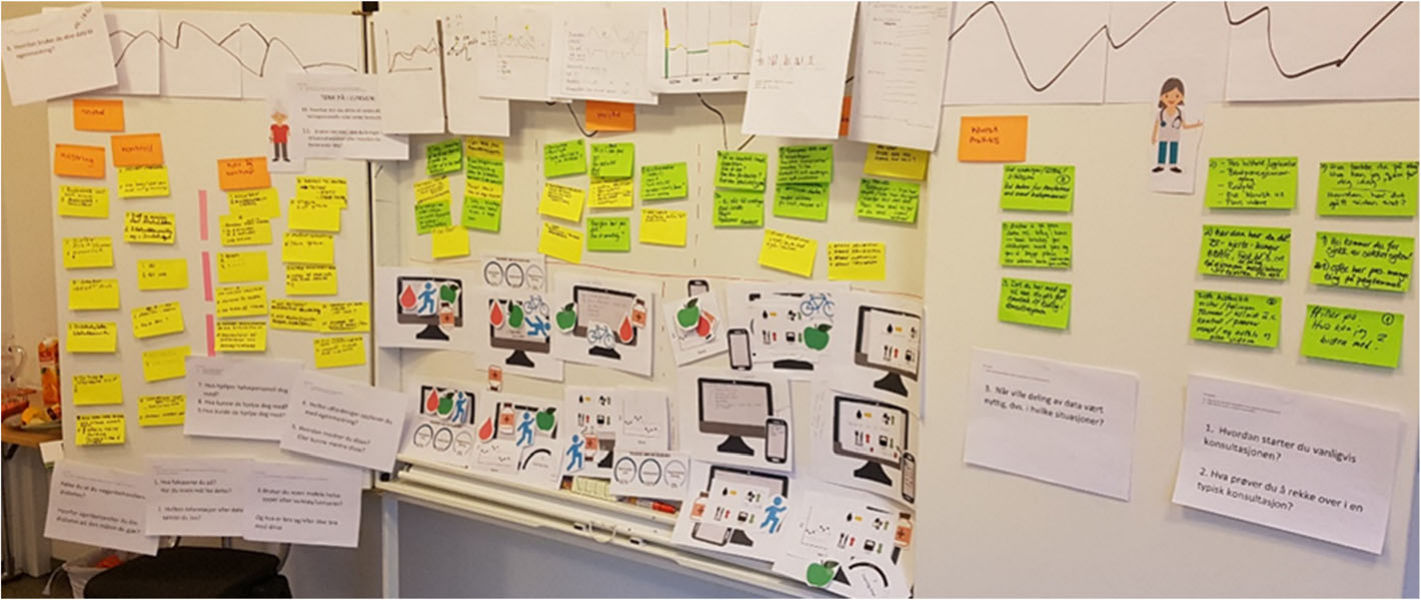
Story-board and post-it notes generated during the first co-design workshop, illustrating the T2D patients’ and GPs’ situations and their expectations of a system for sharing patient-gathered data

Seven individuals attended the first co-design workshop, related to T2D (Fig. 1), and eight individuals attended the second workshop, related to T1D.

While it was not required for participants to offer these information, as the focus was on development of the data-sharing system, some did offer some personal information when asked introductory and ice-breaker questions. The available details are provided in Table 2. HCPs offered only basic information about themselves before offering their opinions of mHealth and data-sharing (Table 3).

**Table 2:** Demographics of T1D and T2D patient participants in both co-design workshops

Diabetes type _Patient#	Gender	Age range (yrs)	Duration of diabetes (yrs)	Reported technology used	Reported self-management foci
T1D_Patient#1	F	40–50	N/A	N/A	N/A
T1D_Patient#2	M	20–30	2	Apps, insulin pen	Physical activity, BG
T1D_Patient#3	M	50–60	30	Insulin pump, CGM, app	BG, physical activity, insulin, carbohydrates
T1D_Patient#4	M	40–50	38	Insulin pen, app	BG, insulin
T1D_Patient#5			N/A	Insulin pen, app,	BG, physical activity, sick days, insulin, diet
T1D_Patient#6	M	60–70	8	Smartwatch, insulin pen	Physical activity, insulin, BG
T2D_Patient#1	M	60+	12	Paper diary, app	BG, physical activity, diet
T2D_Patient#2	M	60+	N/A*	BG meter, insulin pen, paper diary, app	Diet, medications (non-diabetes related), insulin, physical activity
T2D_Patient#3	M	60+	3	BG meter, apps	Diet, physical activity, well-being
T2D_Patient#4	M	60+	N/A**	BG meter, paper diary	BG, input from doctor

*Participant stated “a good amount of time ago”

**Participant stated that they were “in the introduction phase” of their diabete**s**

**Table 3:** Demographics of participating HCPs in both co-design workshops

Provider#	Gender	Age range (yrs)
Specialist#2	M	50–60
Specalist#1	F	60–70
Nurse	F	30–40
GP#1	M	50+
GP#2	F	30–40
GP#3	F	50+

### Main themes identified

Across the workshops, the following three main themes were identified: 1) patients’ and providers’ need for more specific and detailed information in diabetes care 2) mHealth technologies’ impact on patients and providers, with subthemes concerning a) both groups’ use of patient-gathered data and b) roles and responsibilities, and 3) data-sharing, with subthemes concerning a) expectations of sharing and receiving PGD during consultations, b) what and how to share PGD, c) electronic health record (EHR) integration and d) concerns. Because each session focused on allowing the participants to drive the discussion, each theme and sub-theme varied in the amount of feedback participants’ provided. Therefore, for the themes and sub-themes that generated lengthy and diverse feedback, tables are provided for each-sub-theme to summarize and differentiate between responses of each group. Additional quotations from the transcripts, and details about responses for the sub-themes, are provided in Additional File 2.

#### Theme 1: patients’ and providers’ need for more specific and detailed information in diabetes care

At the beginning of each workshop, participants were prompted to describe their overall self-management and clinical practice, respectively. Responses about sub-theme 1A: What and how information is needed are exemplified in Table 4.

**Table 4:** Summary of responses about what and how information is needed by patients’ and providers’ regarding diabetes self-management and clinical practice, respectively

Groups	Codes	Summary	Example quotation
**Participants with T1D**	What information	Answers about specific challenges in their self-management	*“[What is important is] not what we struggle most with on average but what we need to do in specific situations and individual days that stand out as being difficult” (T1D_P2)*
	How information is or should be shared	Answers in the form of recommendations from HCPs about why specific self-management challenges occur and how to respond to them	*“[Most healthcare providers] too far away from the specific situation … You get answers after a day or two … but that is not when I am in the situation … I don’t want to disturb doctors and nurses with my small problems, but maybe they are not so small if we acknowledge what they really are” (T1D_P3)* *“More appointments more frequently … and maybe get more continual information … since the [diabetes] situation changes” (T1D_P6)*
**Specialists**	What information	• To differentiate patients based on situation and needs • To understand patient’s mental state to effectively guide them	*“For example, I cannot expect this one man to get a perfectly controlled diabetes. I would be happy if his hba1c came down to 9%, whereas another patient who is themselves a doctor, I can expect him to have an hba1c around 7% or even below 7% without hypoglycaemia” (Specialist2).* *“Separate the patients in two groups - the ones who have hba1c higher than 8.5 or 9 who are the higher risk ones, [and] the ones with lower than 6–8% who still have problems … different problems” (Specialist1)* *“A person’s mental state and resources, of course … gives you a background for what kind of targets you can expect” (Specialist2)*
	How information is or should be shared	It is the responsibility of the patients to collect and share information as well as provide explanation of their situations.	*“[Patients must] take the responsibility [themselves]” in order for the HCP to be able “to understand diabetes and insulin and how all these things function together” (Specialist2)*
**Participants with T2D**	What information	• Motivation, • To understand how lifestyle choices affect health (i.e. BG)	*“I was better in the starting phase to note down drinks and food … but it has faded, and I don’t today. Need more motivation” (T2D_P2)* *“I have injured knees and shoulders, so motivation is lacking” (T2D_P1)* *“I kind of feel like I don’t self-manage because … I think it goes a bit slow when I test my blood sugar. It’s usually high and it doesn’t really change much … I can’t see what is happening” (T2D_P1).*
	How information is or should be shared	Disease-specific knowledge from HCPs	*“[Healthcare providers] could be more specific. They are pretty diffuse and say “you can do this”, but they need to be more specific and say “you need to do this”, and then tell me the things I need to be doing” (T2D_P2)*
**GPs**	What information	• Information about specific challenges, • To understand and treat all of a patient’s health challenges	*“If [the patients] have reliable information, we use that more than medical history because things happen along the way” (GP1)* *“Patients don’t just have diabetes. Many are mixed with a lot of other things and I feel that can be confusing because they high blood pressure, maybe are overweight, maybe have low back pain, maybe a lot of other things” (GP3)*
	How information is or should be shared	Health measurements and patient recollection/evidence of challenges to then discuss together	*“Things I find important focus on how it has been since last time. Any hypos? Are they in okay shape? Anything wrong? Sometimes I check blood pressure, but not always. I usually check hba1c … then we make an appointment and discuss the plan” (GP1)*

Both those with T1D and T2D had similar experiences with healthcare providers – lack of specific feedback and information. Differences in self-management and care of T1D and T2D were evident in the details, for example, when individuals needed specific support from their healthcare providers. For those with T1D, support is needed when a challenge or symptoms arise because their symptoms and challenges occur more frequently and immediately. However, those with T2D experience more delayed symptoms, making it difficult to identify the cause leading them to need to accumulate information over time and then seek guidance or answers about how those decisions affected their health. GPs and specialists agreed in the importance of specifying their recommendations based on a patient’s situation, but noted that this also requires patient engagement. Specialists mentioned that mental health and a patient’s knowledge and skills affect their expectations of their patients with T1D and how they approach diabetes care. The participants’ background with diabetes care allowed us to identify potential needs for mHealth and data-sharing support for both individuals and healthcare providers during consultations.

#### Theme 2: mHealth technologies’ impacts on patients and providers

As one participant stated concisely, “diabetes doesn’t happen in a container. There are other things around it.” [T1D_P3].

##### Subtheme 2A: purposes of, and challenges related to, mHealth and patient-gathered data

Participants were promoted to discuss how they used mHealth technologies and patient-gathered data for self-management and during clinical practice. Both groups of T1D and T2D participants used their own-gathered data to find patterns by comparing their self-management actions to their resulting blood glucose levels. However, differences emerged regarding what kind of information they aspired to understand, how much data, and over how long a period, these comparisons were made. Responses about sub-theme 2A: Purposes of and challenges related to mHealth and patient-gathered data are exemplified in Table 5.

**Table 5:** Summary of responses regarding purposes and challenges experienced by patients and providers when they encountered or used mHealth devices or patient-gathered data

Groups	Codes	Summary	Example quotations
**Participants with T1D**	Purpose	• To identify similar situations • To identify relationships between parameters, e.g. BG and diet	*“Similar situations … I rarely eat ice cream so I can go back and look at how much insulin I took then and how my blood glucose was after” (T1D_P5)* *“Seeing patterns about what I ate and did in relation to my blood glucose” (T2D_P2).*
	Challenge	Lack of support/guidance to interpret data	*“The lack of support from the healthcare system”, asking “where is the course where I can learn as a patient? I take more responsibility for my own health when using mHealth tools … [and get] a better overview … But even though I know a lot … I want to know more and I want to do better” (T1D_P1).*
**Specialists**	Purpose	For technology to support patients’ self-learning	*“Use of technology needs to create patient action … We want these sort of [patient-gathered] data to be self-learning technology” (Specialist1).*
	Challenge	Limited capacity	*“The number of consultations in our out-patient clinic has increased steadily during the last years. And I remember when I started there almost 20 years ago we had so much more time for patients” (specialist2).*
**Participants with T2D**	Purpose	• To understand long-term effects of lifestyle choices on diabetes health • To spend less time worrying about their health and more time living	*“[I look for] the results for stress level, drinks and such … to find the causes for high blood glucose” over “days, sometimes a month sometimes three months, between the evaluations” (T2D_P1).* *“spend less time and energy on self-management” (T2D_P2)*
	Challenge	• To understand relationships between parameters, • To trust in technology to function properly, • Cost (in some cases)	*“I document blood glucose in the Diabetes Diary app. Plus, I have it on paper too. I don’t trust electronics. I do double” (T2D_P1)* *“I stopped the electronic way because I was abroad and it cost a lot. But I record manually” (T2D_P2).*
**GPs**	Purpose	*Not specifically stated*	*N/A*
	Challenge	Inconsistency in and lack of patient-gathered data	*“They just test three days before, but then stop testing for half a year, and then come back with three lost test-days. Some are testing every day, four times a day … Some have blood pressure monitor at home, that they show me” (GP3)* *“I rarely see [paper] diaries with lots of measurements … many of them have Fitbit but I haven’t seen the results from them” (GP2).*

Those with T1D tend to look at information related to daily experiences. In contrast, T2D requires less frequent measures, which is consistent with both patients and GPs’ focus on longer-term health control and expectation of less data. These differences between patient groups point to how much information either group would gather and possibly present during consultations as well as their driving health goals. It was also evident that the ability of those with diabetes to collect much data has affected what healthcare providers expect of their patients.

##### Subtheme 2B: roles and responsibilities

Within the formal healthcare setting, those with T1D and T2D note that the value of healthcare providers is based upon their ability to understand the patients’ everyday reality of living with diabetes. They also share similar frustration with healthcare providers’ lack of such specific knowledge and answers, when the patient needs it. However, during consultations, the role of authority figure is different in either case (Table 6).

**Table 6:** Summary of which roles and responsibilities patients and providers perceived of one another given the introduction of mHealth into diabetes care

Groups	Codes	Summary	Example quotations
**T1D participants**	Own role	Have control and responsibility for own health	*“You have to take responsibility for the things not being done by healthcare … you have to follow up yourself” (T1D_P3)*
	Specialists’ role	Nurses support patients with answers to specific questions	*“[Want] more specific answers on situations and questions when I am meeting with the nurse. I sometimes have questions about different situations … and two similar situations can become two completely different ones. [And the nurses] never has any good answers” (T1D_P5)*
**Specialists**	Own role	• Advisors • To distinguish between what kind of support different patients need	*“Task is to be advisors. We can’t change anything, we can just give advice. The data by itself needs to help the patients to do the best thing” (Specialist1)* *“We have to start differently and expect differently from our patients. This is about individualization of treatment” (Specialist1)*
	T1D patients’ role	• Have responsibility and are decision-makers for own health • Must be the one to initiate contact with HCPs when needed	*“To make the appointments, and to bring some own generated data” (Specialist2)* *“Be prepared for the consultation. Because we have so little time” (Specialist1)*
**T2D participants**	Own role	Informed data-collectors	*“My role [in sharing data] could be to be more exact in documenting information, such as diet, physical activity … that can help the GP confirm where I am in the process” (T2D_P2).*
	GP’s role	• Interpret patient-collected data • Authority figures, but GPs may not be the best HCP to answer diabetes questions	*“It is interesting … with input from doctor from more examinations and closer follow-up... I miss that, and I am uncertain” (T2D_P4)* *“[GPs] really lack the knowledge in which we diabetics struggle with [because they] do not have enough education to cope with those specific health issues” (T2D_P2).* *“There are also diabetes nurses … they can maybe give more input about what you should do and not do … let the doctor take the more serious, while nurses help along the way” (T2D_P1).*
**GPs**	Own role	• Teachers of patients • To give advice	*“[Patients] are our pupils, and we are their teachers so when they do homework, of course I want to see what they’ve done. And then … I can begin to give some advice” (GP3),*
	T2D patients’ role	Have main responsibility for health	*“You take care of your own disease, not me. I will help you on the way. It is your responsibility, and you have to have some sort of a motivation for it” (GP2).*

Those with T1D appeared to place themselves in the role of authority and decision makers. In these cases, healthcare providers – mainly diabetes nurses - are seen as sources of suggestions and information about unique situations that an individual may face in their daily lives, yet the individuals are the ones to use of the data and make the final decisions about their health. This division of responsibility and roles within T1D care also seemed unanimous and expected amongst healthcare provider. Specialists stated that outside of the consultation, patients were expected to be active in using and understanding the data they generate. While, in the previous sections, those with T2D established that they value mHealth and its ability to help them to better understand their health, in the formal healthcare setting, individuals with T2D place more authority in the healthcare providers. Also, they make a distinction about which healthcare provider is better prepared to answer their specific questions.

#### Theme 3: The data-sharing system

##### Subtheme 3A: expectations of sharing and receiving PGD during consultations

With regards to their expectations of sharing data with their healthcare providers, participants with T1D and T2D were similarly concerned with receiving specific and relevant answers. Just as with the theme of roles and responsibilities, differences between expectations of those with T1D and T2D centered on the level of detailed feedback from their providers, who to contact and overall goal of the consultations when sharing data (Table 7).

**Table 7:** Summary of patients’ and providers’ experiences and expectations of sharing patient-gathered data during consultations

Groups	Codes	Summary	Example quotations
**Participants with T1D**	Experiences	• Without data, feedback is too generic • With data, discussion is more practical	*“[Without data], often I feel like the meetings I have with them, it’s like – “how do you feel” and [I say] “I feel its fine”. I don’t get that much out of [the consultation]” (T1D_P2).* *“[I get] specific tips with things [the doctor] extracts through the data which I don’t feel like I saw myself. I’ve gotten advice that works” (T1D_P2).*
	Expectations	• More specific feedback based on own-gathered data • Interoperability will limited HCPs in their ability to interpret data	*“[Healthcare providers could] Interpret data with the knowledge they have and then give specific tips and feedback about the data” (T1D_P2)* *“Maybe [HCPs] can help me more if they see that there’s a reoccurring problem … if I’m high during the evening...we can try to talk more specifically” (T1D_P3).* *“The [insulin] pump has all this data, so when I come to the nurse she puts the pump into the computer then she runs through and program and sees everything, and … it doesn’t turn into much … with having a lot of data … [its] because of the tools [the HCPs] use” (T1D_P3).*
**Specialists**	Experiences	• Not all patients use, or want to use, these technologies • Some patients do not use the technology as HCPs would like • Those who understand the potential benefit of the technology use it correctly • CGMs and pumps are the most common technologies seen, few apps	*“They can come with all sorts of data, because it’s automatic. But they haven’t made a diary or sort of explained why was it like this, why did I get a hypoglycaemia … saying “oh these are my measurements” and “ohh no, I haven’t looked at them” then it’s so useless” (Specialist2).* *“They check a lot of blood glucose and they actually write it down for me because they realize that when they come with their small booklet then we can talk about it together and see” (Specialist1).* *“When we are talking about new technology, it’s mainly based on CGM. Because that’s the new technology the past 10 years” (Specialist1).*
	Expectations	• Patients will pre-digest data before consultations, then present it to HCPs • Patients who use mHealth are adept enough to use it correctly • Too difficult to understand all of the diverse health technologies	*“Patient X comes in and she has her measured blood glucose … on her device, whether it’s a telephone or not. You get it on the doctor’s screen … and then you see if it’s high in mornings and so on, and you see how much insulin you use. You have the patient already before the consultation – trusting in her responsibility and her interest in doing better” (Specialist1).* *“[Use of mHealth] requires some technological insight and of course some intelligence in a way or - you understand me - stamina” (Specialist2)* *“Less than 50%” of their patients bring their own data to the consultations, either written in a book or* via *an app...[and] I don’t know how many of my patients would like to use the Diabetes Diary app - maybe 5–10% - because it’s too much!” (Specialist2).*
**Participants with T2D**	Experiences	Frustration with GPs not being able to answer specific diabetes questions	*“GPs are busy with work, so … it would be better to get an appointment at the hospital with a diabetes nurse, maybe once a year, and discussed your case with your data. And if you are way off with your values, you could also discuss with a doctor and then come to a conclusion” (T2D_P1)*
	Expectations	Perceives that the GP wants patients to come to consultations with an agenda/questions and corresponding data	*“I think what doctor expects is that you bring your blood glucose measurements, at least from the last week [with] notes about diet, physical activity, [if I] ate too much or drank too much. Compare my own measurements” (T2D_P2).* *“As I see it with the GP, you go to them when you have a specific problem. If you have [an annual check-up] with diabetes, [you are not going because of] a specific problem” (T2D_P3).*
**GPs**	Experiences	• Without specific questions or data, the consultation discussion is “boring” • Wishes for the patient to explain their situation in more detail	*“I think it is a bit boring. “This doesn’t look pretty good, go home and be better”. We need to know how you have been doing, what has happened. That’s what’s going to start a discussion” (GP2).*
	Expectations	• That the patient-gathered data must be easy to understand, will save time and result in specific and realistic goals for patients • Patients and providers will discuss data together	*“[It is possible] if the patient comes with [PGD] and it is easy to understand” (GP2)* *“[Patients need to] understand how to get there. To say getting HbA1c down by doing X. Very specific. In that case, say “you won’t have blood glucose under that and that, and you will walk 5000 steps each day”. Specific feasible goals from day to day” (GP2).* *“What happened to that resulted in these data? What has happened here? Good and bad. Why is it like this?” they could “make a plan to reach a goal - make a decision together … because it is the patient who has to go through with it and follow it up, regardless of what we write... it has to be feasible” (GP3).* *“I think you go through data in together. Look at it together, both and points and trends, both hard data and stories … specific information will save us time, instead of trying to make people tell us” (GP1).*

Participants had experienced the expected benefits of sharing their own-gathered data, i.e. more personalized self-management recommendations. However, even with data, others experienced the limitation of interoperability problems of healthcare technologies. Participating specialists expect that those individuals who use health technologies, including both medical and mHealth devices, pre-digest the data to identify self-management problems before coming to the consultation. However, specialists also explained the diversity of experiences and expectations in their clinical practice, including the fact that many either do not use these technologies or do not use them optimally.

The expectations and experiences of those with T2D and GPs reflected a different dynamic between individuals, the technology and their providers than those with T1D and specialists. While those with T2D did want specific answers, they were first and foremost concerned with the concept of communication and responsibility; when to communicate and with whom, in order to receive the type of answers they wanted. Participating GPs also acknowledged the challenge of providing specific feedback to their patients in the absence of data. Like those with T2D, GPs were also interested in communion but more specifically, shared decision-making and believed that specific data would lead to specific and realistic goals for the patients.

##### Subtheme 3B: what data to share and how to display it

Referring to their own developed paper prototypes during the joint session, participants were able to explain how their ideal system would function to generate a discussion (Table 8). For quotations that detailed both what and how the data should be displayed, cells within the table are merged.

**Table 8:** Summary of patients’ and providers’ ideals about what and how a data-sharing system would present patient-gathered data during consultations

Groups	Codes	Summary	Example quotations
**T1D participants**	What data to share	• Indications of specific problems in their self-management • Concerns about what data to share	*“We could get a sign on the graphs … maybe statistics on how the blood glucose is … in the evenings or afternoons” (T1D_P5)* *“What I need is different than what you need as a doctor” (T1D_P4)* *“Summaries of my every-day [data] in such a way that we together can discuss where the problems are” (T1D_P4).* *“I have a lot of data and my ideal situation is that I get a mail from my nurse saying I want your data, or a reminder. Or I upload my data in my program and share with my nurse and then I get the question “Can you note this week what you put of insulin in the given period and then I get the data from you” (T1D_P3)*
	How to share patient-gathered data	• Summaries • Graphs, e.g. showing trends during different times of day • Symbols to indicate change of a data type over time • Provide specific data as requested by healthcare provider	
**Specialists**	What data to share	• Fluctuation and trends • Indication of what patient’s problem/challenge is within the data • Representative data-sets	*“First, I would like to see the fluctuation [of blood glucose] over 24 h - it’s the most important for me. Then have a look at some data because there was something special going on” (Specialist1)* **(**Fig. 2**)***.* *“An intensive period [worth of data], maybe some days or weeks before they come to me, because I want to see variation. And document pretty carefully … Then we can see the context … So these very like, these short, tiny, detailed periods is very valuable for me even if it’s not representative for the long life” (Specialist1).* *“The last week or 14 days … where you can see meals, calibrations - to see that you calibrate correctly - physical activity and illness … to explain why you are high the whole night, and of course insulin doses. Additionally, if the algorithm can pull statistics and say “ok, you are always low after correcting extra” or such things” (Specialist2)* **(**Fig. 3**)***.*
	How to share patient-gathered data	• E.g. algorithm or statistics • Ability to choose which comparisons to make within the data provided	
**T2D participants**	What data to share	• Overview of own situation • Status of self-management habits, i.e. each data type gathered • Concerns about what data to share	*“Having summaries of the data, and then [you can] click on blood to get [more details] … what you’ve done that day and time and all. Everything in a submenu of the main” (T2D_P2)* **(**Fig. 4**)** *“I can collect irrelevant data - I can gather data about my own situation that may not be relevant for doctors” (T2D_P4)* *“You have green and red and yellow zones. I might not need all the values, but you could see if you are safe or not. Like the weight it is pretty much too high all the time. Physical activity is maybe not so good.... And then you could choose, and get out the exact values, as a table” (T2D_P3).* *“A diagram with levels - level for goals, level for what was completed.... With remarks and blood glucose data and diet” (T2D_P2)* *“A bar-graph … plotting blood glucose, exercise and wellbeing. Nothing more” (T2D_P4).*
	How to share patient-gathered data	• Diagrams or graphs with colours or indications of change • Comparison of self-management habits vs. goals • Ability to choose which data-types to explore from a summary	
**GPs**	What data to share	• Challenges or issues within a patient’s self-management habits • Detailed data for challenges	*“I tried to get in everything at once [to] see a correlation if you have [different PGD] together … You won’t bother to plot it every day, but rather have a marker of some sort if it was something special...like if [the situation is] suddenly changing- the values go up or down, their health situation is getting worse or something- it could be okay to have more values, to see what is actually happening” (GP1)* **(**Fig. 5**)***.*
	How to share patient-gathered data	• Summary via, e.g. Graphs • Indicators to show if “something special” (challenges) happened • Correct and representative data	*“Type of compressed summary...Instead of having to look at a thousand measurements” (GP3)* *“If you get graphs and stuff it is easy to relate to and you can get quick glance of what has changed. But if you get the whole [data set] in reverse and just scroll and scroll, then it’s not very useful” (GP2).* *“With physical activity, having it correct, so maybe step counter for example. It says something about changes. Useful if you have these watches. They are not necessarily very reliable, but it says something about your development. Instead of you saying you went for a walk, or half an hour, which doesn’t really tell me much” (GP1).*

Participants’ comments converged on the end goal of information exchange - generating discussions. Both patients and providers acknowledged that each had relevant and desired information to exchange, and an opportunity to do so with mHealth, that was not commonly used at the time. A comment from one specialist summarizes what all seemed to hope for from a data-sharing system – to facilitate information exchange; “One thing is data sources another thing is information. Because the information is generally the communication with the patient at the site there and then” (Specialist1). However, both those with T1D and T2D independently identified a challenge that should be addressed within this type of information exchange.

**Fig. 2:**
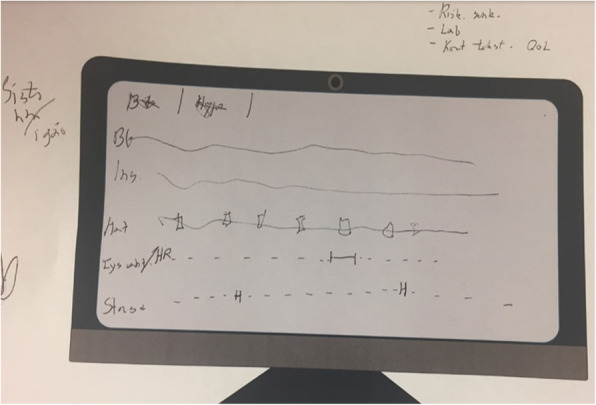
Specialist 1’s paper-prototype for an ideal data-sharing system display

**Fig. 3:**
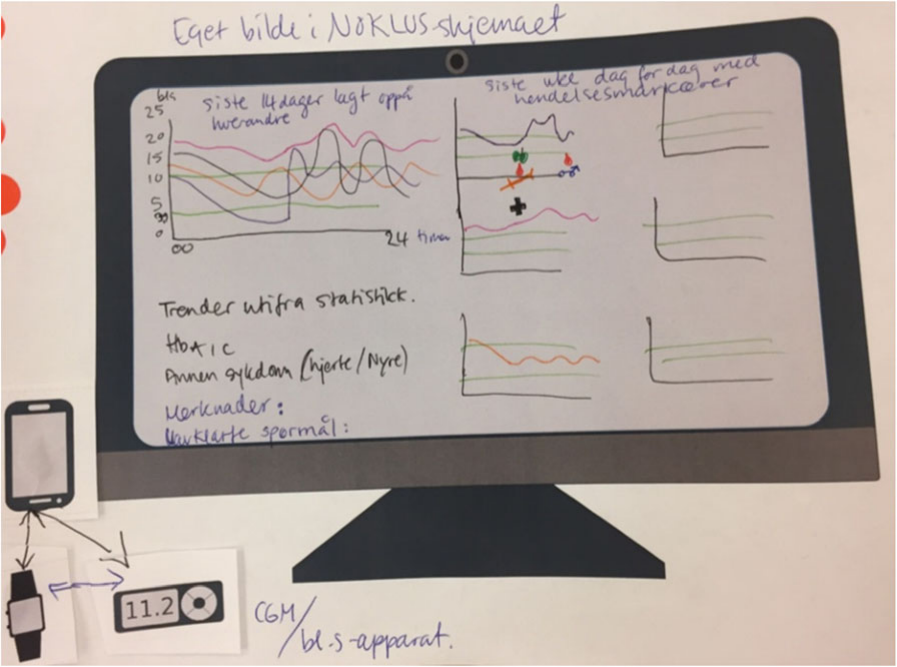
Specialist 2’s paper-prototype for an ideal data-sharing system display

**Fig. 4:**
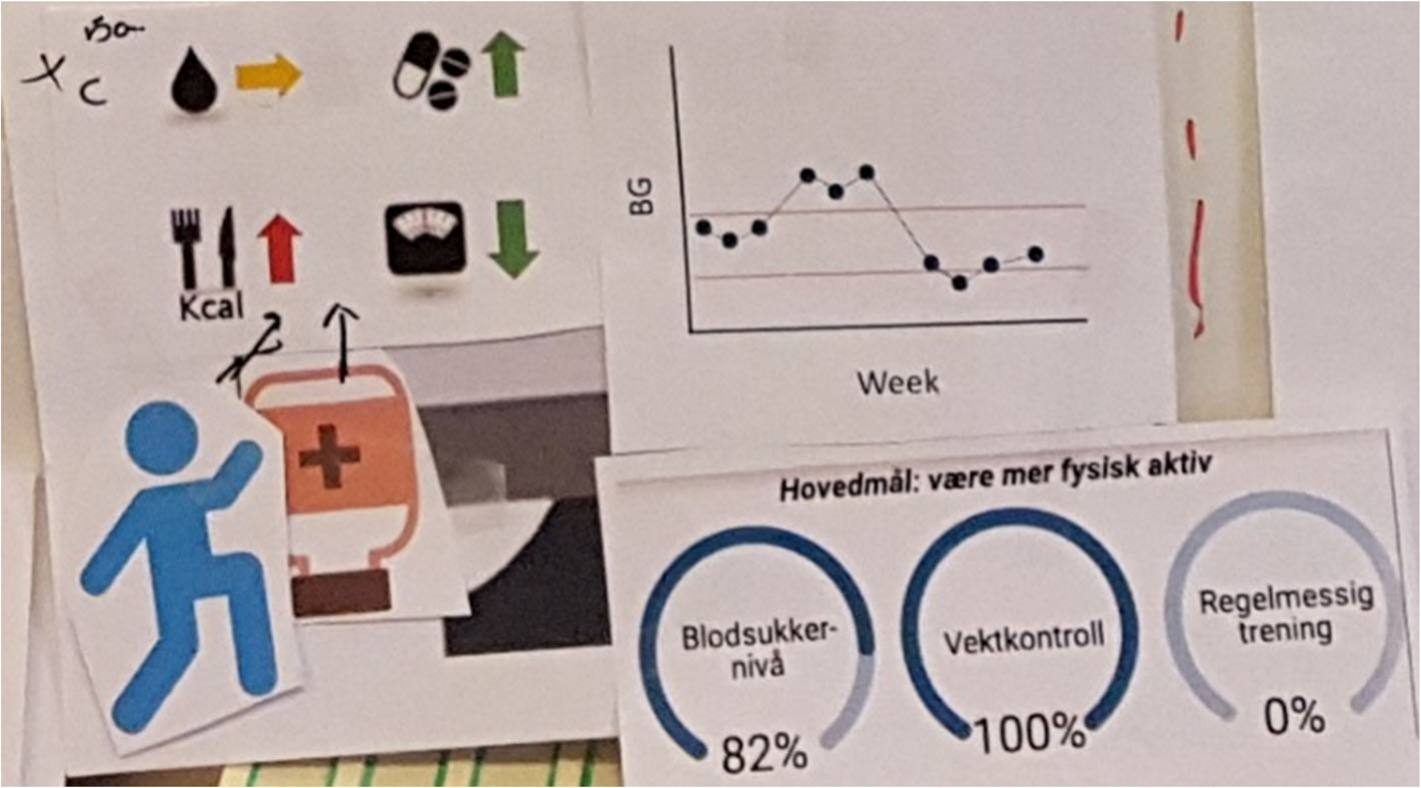
T2D Patient2’s paper-prototype for an ideal data-sharing system display

**Fig. 5:**
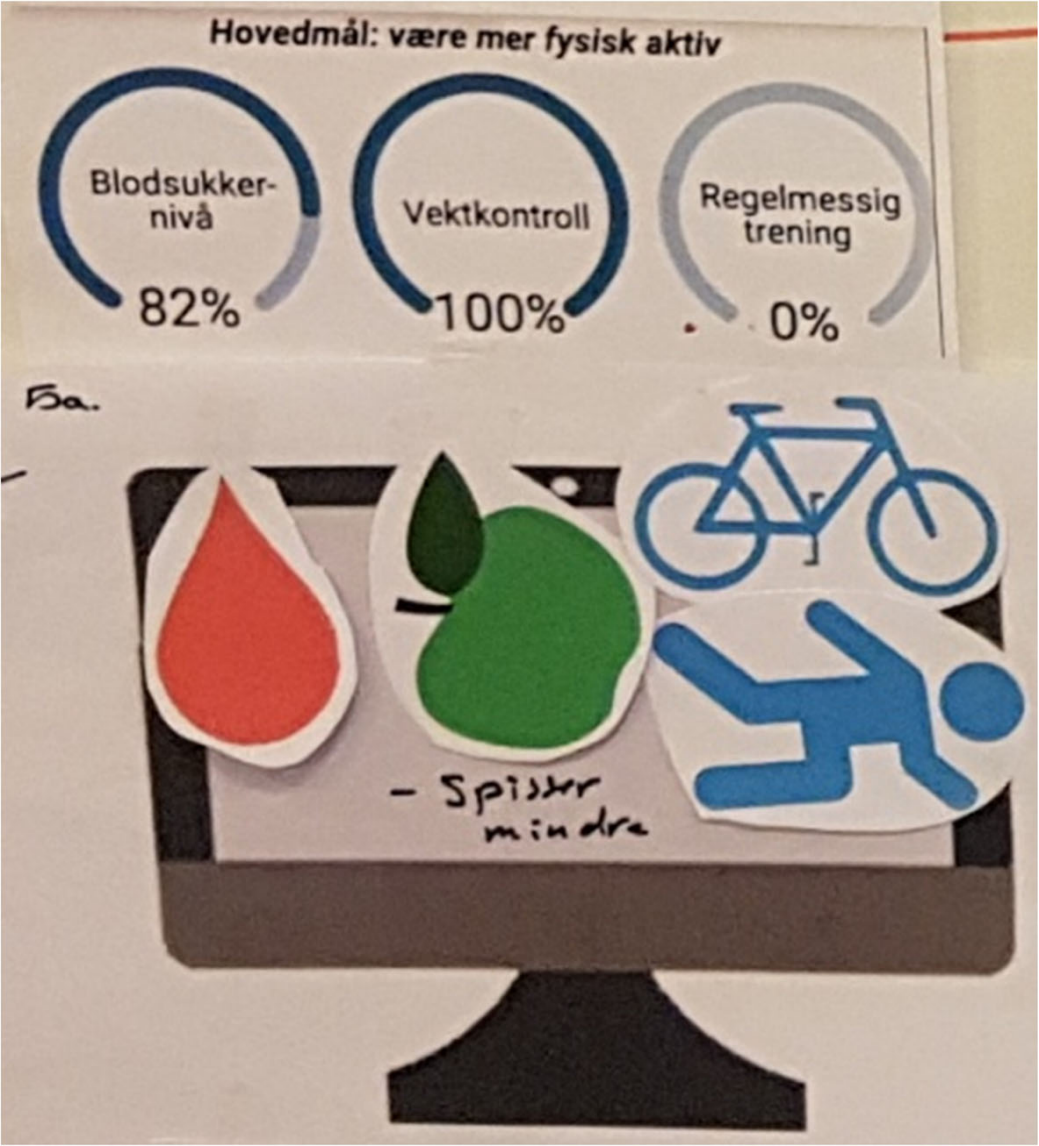
GP1’s paper-prototype for an ideal data-sharing system display

Suggestions from both patients and providers were similar in that they would like a system that summarized the PGD, with the option of choose which data to explore further, if trends or outlier points were identified. Those with T1D wanted answers about specific challenges that they experienced and documented. Those with T2D wanted an overview of their progress and feedback about how to progress. One GP expressed the value of a diverse data-set while another expressed that, for some parameters, exact values were not as important as bringing correct and representative data. Figures 2, 3, 4 and 5 illustrate examples of paper prototypes designed by the participants.

##### Subtheme 3D: electronic health record integration

Specialists and GPs preferred different ways of accessing and integrating the data into their everyday practice (Table 9).

**Table 9:** Summary of responses to perceptions of mHealth and patient-gathered data being integrated into healthcare providers’ electronic health record (EHR) systems

Groups	Codes	Summary	Example quotations
**Specialists**	Preferences	• Automatic data transfer • Visual summary of specific data types within patient-gathered data	*“Automatically getting the continuous glucose values for the last week, into my electronic diabetes journal system... and the use of insulin or automated data easily, visually presented” (Specialist1)*
	Risks	• Data-overload • Capacity of personnel and resources • Personal liability of not identifying indicators of dangerous habits and symptoms	*“The other thing that comes to my mind when you say [integrating technology] is “Please stop it!” because I if you are the patient and I get your data continuously for your whole life on my screen, then I am responsible because if something happens to you, if you go into your car and have a traffic accident with hypoglycaemia it’s my responsibility because I should have seen that last week you had several hypoglycaemias … but we do not have the resources for this” (Specialist1).*
**GPs**	Preferences	• While prefer no integration, alternatives could include automatic and simple data-transfer that do not require the provider to perform additional tasks • Rely on entering own notes into EHRs about a patient’s status	*“We don’t need to load [PGD] into the EHRs, because there are many problems and overload of information. And, why should we keep it?” (GP3).* *“If we would to have it on our computers, partly* via *a journal. Not extra software! Then [the patient] can have [their] phone, plug in USB, and I have it, okay, we could do that … a compromise - But not one manually!” (GP3).* *“Instead, I prefer] to type [notes about PGD] myself … write it short. Reminder [to focus on this] for next time” (GP3).*
	Risks	• Data-overload • Overloading the provider with additional tasks	“*It’s always a chance of overload … a whole lot of data. We can’t relate to it” (GP1)*

Subtheme 3E: Concerns.

Despite participants’ optimism and the potential that they saw with sharing PGD, providers consistently noted their concerns (Table 10).

**Table 10:** Summary of responses to perceptions of mHealth and patient-gathered data being integrated into healthcare providers’ electronic health record (EHR) systems

Groups	Codes	Summary	Example quotations
**Participants with T1D**	Concerns	• Data overload • That healthcare providers still would not be able to use data to generate personalized recommendations	*“Giving up all of my blood sugar measurements is very much information” (T1D_P4)* *“[I shared] a lot of data … I got little use out of meeting the diabetes nurse...Last time she said it wasn’t much she could help me with” (T1D_P3)*
**Specialists**	Concerns	• Priorities of healthcare providers would be hindered • Healthcare providers’ capacity, i.e. time required to use and knowledge about how to use technology	*“The CGM technology is very good, but our nurses – it means that 80% of their time is working with the functional learning patients and [complaints about] “it doesn’t work”, “how can it do it and change”. So instead of actually talking to the patients about “how are you” – we are dealing with problems about “I can’t fit it” (Specialist1)* *“10% [of patients] use the CGM. Then those patients get much more consultations with the nurses because they need to be taught the CGM and they need follow-ups. So this small group … maybe they use 80% of the nurses’ time” (Specialist2). Also, continuous data transfer potentially meant the need “to have a diabetes nurse continuously, 24 h-a-day, checking on continuous glucose monitors (CGMs), like we do with hospital patients. We don’t have resources for this” (Specialist1).* *“There is a lot of different technology now. It is Freestyle and it is CGM and it is 640 G and Freestyle Libre. Of course patients are oriented about this because they talk to other patients, search on internet and so on. But the doctors have very little possibilities … so, we have little time to learn these new systems and to understand how to optimally use them” (Specialist2).* *“During this 30mins where I am also supposed to have nice communication with the patients and also do blood pressure and check their feet and check if they have been to the eye doctors, checking and requiring lab measurements and prescribing insulin– I need to get these data in. That’s a problem of time. The other thing is the problem of methodology of how to get the data presented in a way so that it is not time consuming for me” (Specialist1).*
	Alternatives	• PGD could complement and be used together with EHR systems, if it were to be integrated automatically	*“I work in an [electronic health record system] so there we have a lot established already. And what I want is a new screenshot showing blood glucose...and the insulin. It has to happen automatically, either with pen or pump. And then something about physical activity. And then something [about] food, and short [remarks] about stress and questions for the doctor … And then we look at it on the screen together. Then it is easier to see and explain, and with things already in [the electronic health record]” (Specialist1).*

As mentioned above, specialists were specifically concerned with healthcare service priorities and resource management. Specialists were also concerned with how and where they should go to learn how to use these technologies, because they lack the time and support to engage with these types of new medical and mHealth devices technologies. Those with T1D shared the providers’ concerns of data-overload. Both healthcare providers and patients expressed a desire to share relevant and discussion-worthy information during diabetes care, but these barriers highlighted reasons that some are reluctant to integrate PGD from both medical and mHealth devices.

## Discussion

### System design

The co-design workshops focused on options for integrating mHealth as a supportive tool for diabetes care – designing a system for sharing patient-gathered mHealth data during consultations. Common design features that were identified included a) the presentation of PGD in a summary on the first screen of the system, with the option to select more detailed views and combinations of information on subsequent screens, b) graphs and charts were popular choices for visual representations, especially when comparing different data types, c) visual indications of change such as arrows or symbols related to each data type based on desired and undesired clinical values, e.g. blood glucose values in high (yellow), acceptable (green) or low (red) ranges, d) presentations of data that is relevant to the patient and e) efficient to use. While both those with T1D and T2D believed that sharing data remotely or before the consultation would allow them to receive answers and guidance during challenging situations and save time for both patients and providers, most providers were sceptical of this idea noting that patients must be present during the discussion in order to share and explain their data effectively. With these design features, both parties would be able to choose which data to look at, and then agree upon feasible solutions together.

These design features support the concept of “shared-decision making”. While this term was meant to refer to patients and providers discussing and sharing the responsibility of deciding the best course of action for both self-management and medical treatment options together [20], much of the literature refers to HCPs making the final decisions in a “paternalistic model” [29, 30], have cited the challenges of or referenced the lack of specific suggestions for how to achieve this ideal [31, 32]. Even when shared-decision making is used in its truest intended way, it still faces challenges such as patients’ lack of understanding of their disease and the providers’ unwavering focus on clinical measures [33]. The results of these workshops suggest that patients and HCPs see that potential collaborative point between their areas of expertise – providers’ medical knowledge and the patients’ mHealth self-management experience an PGD– can lead to true shared-decision making and, subsequently, feasible health goals for individuals.

### Collaboration and understanding

The shared aim amongst patient and healthcare provider participants of displaying these data was to facilitate discussion and shared decision-making. Patients and providers independently and consistently described the value of discussions, exchanging valuable and useful information and for improved communication, not just about the data itself but about expectations and intentions. For example, both those with T1D and T2D wanted to know which data healthcare providers were interested in or needed in order to provide specific feedback and recommendations. While patients hoped that providers could relate to and interpret PGD, providers were quick to explain that it is an unrealistic expectation because the healthcare system does not provide resources to teach providers about how to discuss the various mHealth technologies in care practice.

Participants also expressed an understanding of their counterparts’ situations within diabetes care in general. For example, those with T2D understood that GPs may not be the only, or even the most knowledgeable, source of answers for their diabetes-specific questions. This was expressed with empathy, not judgement. Instead it prompted discussion about realistic alternatives such as going to visit hospital nurses or reputable internet sites. Specialists were particularly concerned with understanding the unique situations of their individual patients. While in some cases their comments were not directly related to the question being asked, it forced us to take a step back in the discussion and understand the reality of diabetes care. For example those with T1D, where one specialist urged us to keep in mind that treatment is about the individual person and their specific situation - a concept which should be more prominently addressed in our mHealth research; addressing those with T1D as a group is not actionable given the unique needs of each person. The other specialist emphasized that providers need a comprehensive understanding to effectively guide an individual, i.e. understanding their mental state, resources and intentions in order to generate a realistic goal for their diabetes. A participant with T1D also reinforced this from the patient perspective by explaining that they would rather have a conversation with their HCP about which data to share in relation to a certain situation so that the consultation could be more productive and targeted.

It is also important to note that the participating individuals with T1D portrayed the need for data-sharing as very straight forward – seeing the situation from the perspective of someone who already is familiar with, and uses, medical and mHealth technologies; i.e. they present their data and the healthcare provider can identify patterns. However, participating specialists made it clear that their perceptions and expectations of sharing data during consultations is much more complex. While some patients can come with a well-prepared agenda, providers also have to prepare to relate to those who only use paper diaries as well as those who try, but do not manage to use the technology as specialists would hope.

### Data sharing and information exchange

Specialists were very aware of the impact of accurate and complete data sets because collecting data is useless if the user is unable to determine meaning from what they measure. They expressed several times that each decision about a patient’s case not only had to be informed by their sense of the individual’s personal situation, e.g. other responsibilities in their life and wellbeing, but also the accuracy of the representations of their diabetes health, e.g. blood glucose levels in relation to insulin doses. GPs, however, were not as concerned with where the data came from as expected. While they did emphasize that the data was representative of the patient’s situation, because, as some explained, they did not intend to alter medication or clinical treatment plans based on this data, the exchange of information was more important. Instead they believed that they could use PGD as an indicator for the patient’s progress and a basis for which patients and providers could together develop self-management recommendations.

A significant distinction between the meaning of “data” and “information” emerged from these discussions. Data is useless on its own. Individuals need to have a purpose, intention and questions in order to direct what data to collect as well as how much and what information, evident from the whole collection of data types, can be identified and presented to their healthcare provider. Healthcare providers may be interested in specific data points when “something special is going on”. However, again, participating providers believed that individual data points, or even a collection of one data type, are useless without context.

### Issues that data-sharing can and cannot solve

By comparing participants’ backgrounds, i.e. general self-management and clinical practice experiences and needs, and their ideas about sharing PGD through a dedicated system we were able to generate a better understanding of what they believe can and cannot be addressed, let alone solved, with sharing data from mHealth devices. While the primary aim was to gather input about the design and functionalities a system should have, participants provided additional information about issues surrounding the use of the system. Especially those with T2D expressed that they often did not know why their blood glucose values were changing so drastically. This was an example of a solvable issue because their ideal solution was that a data-sharing system could not only identify a patient’s challenge areas but correlate the concerning blood glucose values, for example, with their food and medication. Issues that needed to be addressed before such a system could even be realistically implemented were mHealth technology training and support for healthcare providers. Both specialists and GPs expressed their limited knowledge and frustration with not having the resources they need to become aware of or optimize use of mHealth and PGD during clinical practice. For example, specialists repeatedly emphasized their concern about resource management, when technologies required nurses to provide more time and support for a small group of CGM users, and technology training in general, because there are too many different types of technologies to familiarize themselves with.

### Proposed data-sharing system vs. state-of-the-art

We aimed to address what it would take to make the collaboration between patients and healthcare providers using PGD possible and useful for all users. Some of the unique design ideas and purposes for the system that resulted from these discussions were the overwhelming agreement that the system should generate discussions, and more importantly, shared decision-making. The system should be flexible and present an overview of patient-relevant data, and give the patient-provider team the option of further exploring certain data at their discretion. These options and intentions differ from many commercial options or other tested interventions available at the time. Typically, the responsibility and ability to interpret the data and make decisions is one-sided - either skewed toward patient self-management, such as apps found on app stores, or clinical monitoring and oversight of only one parameter such as CGMs [34]. For example, an individual with T1D can use an app to track how each type of food affected their BG levels to meet their goals, whereas an HCP may prefer to see summaries of data such as medication use and response, which can then be compared to lab results. However, participants of these workshops agreed that the potential benefit of using a data-sharing system that would allow both parties to explore the data together, would be to foster mutual understanding and discussion of the data, which could lead to feasible recommendations. The presented users’ feedback support the notion that patients and providers working separately, e.g. with separate agendas for the consultation and poor communication, is not as effective as identifying common needs of both parties and designing systems to support those.

### Reflections on the research method

With respect to the research method itself, it is important to note that these presented results highlighted a significant difference, and challenge, of mHealth research compared to traditional research. Traditional research on medical tools and services follows a thorough, focused and lengthy process. Spending much time on these interventions options is expected and healthcare providers, thanks to the validated and trusted methods of inquiry, accept the results. However, research on mHealth tools and services requires a more user-involved, comprehensive and rapid approach. It calls for not only validation of the technology – which still lacks a standard process, but at the same time, the validation of feasible options for integration into medical system workflows. Therefore, we as researchers must re-evaluate how best to perform research that answers traditional questions, e.g. hard health outcomes, as well as those that are unique to mHealth and personal health alternatives, e.g. ways of gathering and displaying data that both healthcare providers and patient, as experts in their own health, can understand. This includes taking advantage of new resources, e.g. expert patients in mHealth and social media, and more actively collaborating with healthcare authorities and organizations to determine feasible health service options to support mHealth integration for both patients and practitioners. Many co-design workshops do involve patients and HCPs. However, they do so most commonly in separate sessions [35]. In research practice, the interpretation of the resulting participant feedback, often would have to be inferred rather than explicitly stated. In other words, there is usually limited or no possibility for participants in different groups to correct one another’s assumptions. We hope that by demonstrating how patients and HCPs can discuss solutions together, we can encourage others to use the EBCD method more in the mHealth and personal health field.

### Lessons learned

With regard to the methods and approaches used to conduct these co-design workshops, we have generated a list of “lessons learned” (Table 11). Planning of the workshop sessions and activities were generated iteratively over months to ensure that all participants felt prepared and safe to share their perspectives and that we as a research team would receive the feedback necessary to design an end-user-based system for sharing data. We experienced the need for a research team to be flexible, inclusive and have an open agenda when inviting end-users to participate in directing the research.

**Table 11:** Lessons learned about conducting a co-design workshop between individuals and their healthcare providers

Aim	Lessons learned	Recommendations
1. Address topics relevant to the design of a data-sharing system.	Participants have their own agendas when participating in a workshop, e.g. specialists spent more time explaining the situation in their clinics and their views of what patients need in general, than expected and often did not respond directly to the question asked.	Plan for participants to take time to explain their situation. This provides more context for their perceptions and expectations of the situation, allows the research team to better understand their needs, and may provide additional and unexpected relevant information.
2. Explain intentions, e.g. explain how to use participants’ feedback	Know your audience - What you see as important to the core purpose of the project may not be relevant for the participants.	Do not overwhelm participants with information, especially at the beginning when their priority is to get settled in and comfortable. Test out your explanation on someone completely unrelated to the project, e.g. a family member or friend, and ask that they point out the confusing or unnecessary details.
3. Encourage participants to produce as much input about their needs and ideas as possible.	Engaging and creative activities were planned based off of research and online “toolkits” available from several difference organizations. Despites attempts to make instructions as straightforward and clear as possible, participants felt the need to clarify several times because the instructions were either too detailed and complicated or not understandable.	• Use other researchers or staff in other fields, e.g. product development, as resources for activity ideas • Participants may have a different interpretation of the instructions or may miss instructions, in which case it is best to adjust yourself as a researcher to their interpretation instead of trying to correct them as this may be discouraging
4. Create a comfortable and inclusive atmosphere to bring forward honest feedback	We posted signs and reiterated verbally that there are no small or silly comments; all insights and feedback would be welcome. Disagreements were of course welcome but we encouraged respect in the verbal discussions.	Before the workshop, reinforce within your team that this is about the participants’ experiences, not about your own assumptions or preconceived notions of what is happening or, especially, what should happen. Do not take sides if there is a disagreement but encourage participants to explain - ask “why do you feel that way?” or “why do you believe that”.
5. During the joint sessions, ensure that both patient and healthcare provider participants feel comfortable and safe to share their opinions, despite the difference in perceived “authority level”.	We expected to need to reiterate that everyone’s opinion is their own and should be respected. However, possibly due to the less hierarchical cultural structure in Norway, we did not need to reinforce this concept. Participants were respectful and listened without having to be directed.	• Make sure that none of the participating healthcare providers were the clinicians of participating individuals with diabetes. • During the lunch break between the separate morning and afternoon joint sessions, invite all participants to eat together. • Suggest ice-breaker activities, within and between groups?
6. Creating an engaging and creative atmosphere	We chose large rooms and posted the three situations that we aimed to understand (self-management, clinical practice and consultations) on wall-sized poster boards as visual aids. These included pictures and space for participants to draw, write and tape their ideas to.	Introduce each situation and allow participants to familiarize themselves with the posters before starting the activities. Allow them time to brainstorm and encourage physical interaction with the visual aid materials. If participants know what is planned, they can mentally prepare themselves for the day, e.g. develop ideas throughout and know what is expected of them.
7. Allow for the participants to drive the conversation and tell the research team what they need and ideas for the systems’ design	Some participants seemed unfamiliar and uncomfortable with suggesting creative solutions for a future system. Instead they wished for us to present prototypes and then form a discussion based off of existing ideas.	• Expect that different participants have had different history with workshop activities and different expectations going into the workshop • Clarify the expectations of the researchers and participants at the beginning • Participants could also help to plan the workshop and select activities that their believe will allow them to most accurately and completely share their opinions

### Study limitations

#### Geographical region

Limitations of these workshops resulted largely from the convenience sampling from a specific geographical location – Northern Norway. The relevance of this is that the typical culture of the medical system is less hierarchical. This can sometimes extend to the relationship between patients and their healthcare providers. The consequence is that the use of a joint session in the co-design workshops and gathered feedback therein may not be representative of the type of feedback, e.g. the unabashed correction of assumptions, that could be gathered in other cultures or geographical regions.

#### Gender balance

Another limitation was the lack of gender balance amongst our participants. The relevance of this is that, in general, there are differences between genders with and without the use of technologies. These differences stem from their daily responsibilities and cultural roles that research should be addressing and that impact the outcomes and application of scientific findings in healthcare practice [34]. While we aimed to recruit equal numbers of each gender, few female or non-gender-binary participants expressed interest in participating, e.g. during the T2D patient session in which there were only men. The consequences of this are that there was an overrepresentation of suggestions about how technology should function that suit men, e.g. the ability to collect and share types of data that may be more or less important to other genders. To ensure more balanced participation in future studies, we could allow for a longer response time during the recruitment process, and/or advertise the study in different media.

#### Participants’ level of technology experience

The convenience sampling also relied on recruiting patients who used the in-house developed Diabetes Diary app and were therefore already engaged in mHealth for diabetes. The relevance of convenience sampling for mHealth studies is to recruit those who have experience and therefore experience-based suggestions for how to address the call for mHealth integration into clinical practices; such a group would be likely to consider sharing their app data with their HCPs and would be more likely to know what they would want from a system designed to do so. However, we do acknowledge that these participants were not representative of all patients with diabetes. As the specialist participants echoed, they only meet a small percentage of patients who use medical devices and mHealth technologies. The consequence of this is the potential to widen the digital divide by focusing on further development of modern technologies instead of focusing on how existing technologies can be more inclusively developed and supplied. In the future, all interested and eligible (18 years +) parties could be included to ensure that feedback about mHealth represents not only additional and advanced functionalities but also improvements on existing functionalities to lower the barrier-of-use and increase the benefits of personal technologies for diabetes self-management.

#### Focus of the discussion guides

Further, discussion guide questions focused on data-sharing, use of mHealth and healthcare consultations, not on the demographics of the participants. This led to an incomplete data set, i.e. lack of information about duration of diabetes, exact age, HbA1c, education and other potentially relevant factors. While the primary focus of these workshops was to explore the impact of participants’ experiences and preferences on the design and potential use of a data-sharing system, the consequence was a lack of consideration for what younger vs. older individuals would need from such a system or how they would experience sharing their data with healthcare providers. This can be overcome in future studies, without affecting the workshop time, by the simple addition of a demographic survey at the beginning or prior to the workshop start, perhaps as a part of the informed consent process.

## Conclusion

Those related to T1D care emphasized the need for a system that identifies instances of health issues from individuals’ registered data, facilitates patient-provider discussion, fulfils the information needs of individuals for their self-management and makes it easy and efficient for healthcare providers to view the same data in different ways, e.g. reviewing different time periods or combining different data types. Participants related to T2D care expected that mHealth technologies to motivate patients to track their health and be able to learn more effectively and direct the consultation conversation in a more proactive way. Both those with T2D and GPs hoped that sharing this much more representative data during consultations would provide evidence of trouble areas in the individual’s self-management that they could both discuss and find solutions for, together.

To benefit both of these end-user groups, the system should structure the data in a relevant and usable way, and be flexible enough to present different levels of information, i.e. summarized and in-depth, and be understandable for both patients and providers in order to generate collaborative and tailored discussions. This argues that there should be a single flexible systems is influenced by the healthcare providers’ preference for fewer additional technology solutions and the fact that some individuals with T2D also visit HCPs in the hospital, not just those with T1D. Specialists and GPs agreed that they would prefer not to install, and have to learn, yet another technological system in their practice.

To address healthcare providers’ concerns of their own preparedness and workload capacity, healthcare systems should consider developing support services and resources surrounding mHealth and PGD integration, such as topic-specific education. The verified feedback from these co-design workshops have demonstrated the importance and value of including both patients and healthcare professionals in designing a system for integration of PGD during consultations.

## Supplementary Information

**Additional file 1.**

**Additional file 2.**

## Data Availability

Due to the small population from which the participants were recruited, we believe that sharing the transcripts would be exposing too much identifiable information. Therefore, we will not be making the data openly available. However, as an alternative, we have added Additional File 2, which provides curated quotations from the transcripts that may be relevant for fellow researchers, but were not reported in the main text or directly related to the design of a data-sharing system.

## Abbreviations

mHealth: Mobile health
PGD: Patient-gathered data
T1D: Type 1 Diabetes
T2D: Type 2 Diabetes
EBCD: Experience-based Co-design
HCP: Health Care Provider
GP: General practitioner
EHR: Electronic Health Record
CGM: Continuous glucose monitor
