# Supplementary material for: How mHealth can facilitate collaboration in diabetes care: qualitative analysis of co-design workshops
Source: BMC Health Serv Res. 2020 Nov 30;20:1104. doi: 10.1186/s12913-020-05955-3 (PMC7706243; doi:10.1186/s12913-020-05955-3)
Supplement: Supplementary file 1 — Additional file 1. [file 12913_2020_5955_MOESM1_ESM.docx]

# Additional File 1. Discussion guide for the Co-design Workshops

*The Full Flow research project*

| **Aim (A) and Purpose (P)** | **Questions/activities** | **Practicalities/instructions** |
| --- | --- | --- |
| **Patient session (9-12)** | | |
|  | Welcome & Ice breaker: Year of the Coin | - **Welcome** & return/fill in signed informed consent forms while fill up coffee/tee - **Introductions** of everyone by first name (name tags)   - Describe research team roles so participants are aware   - Discussion moderator (EÅ)   - Agenda facilitator (AsGr)   - Observers and helpers (MB and AG) - Ice breaker: Year of the quarter (tell what you were doing during that year)   - 7 coins, one for each patient participant and researcher |
| **A: Common understanding of what we want to achieve from workshop**  **P: Working within the scope of the project without limiting creativity and input** | Description of project purpose and aim | - - **Introduction to the mHealth environment:**      - We are speaking about patients in general - the average patient may not have so much experience with technology but are interested in using it therefore we must address their needs as well.     - So, participants can educate us about what is important, what works for them and where they need more assistance/support in managing their diabetes.   About the research project:  Mention briefly the aims and actors involved, and the main test we aim for.   - - **Purpose:**      - Want to know how mHealth and patient-collected data can be used in clinical consultations     - Help patients and clinicians make better plans for managing their own diabetes     - All of this will help design a system where you and your clinician, together, can view the health data that you collect yourself.   (Visually displaying the ideal situation that we hope to achieve – put this in the center panel of the landscape)  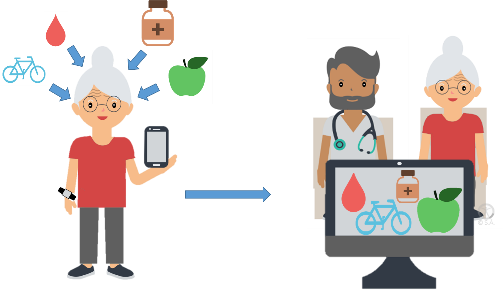   - **Overview of the day and agenda:** - So for today, we will get to know how you interact – or wish to interact - with your apps and self-collected data and then, in the afternoon together with clinicians, what you would need from a system that helps you to share and discuss your data during consultations |
| **A: Common understanding of how to conduct one’s self during the workshop**  **P: Encouragement and comfort of ALL participants, establish safe environment to share own ideas and receive others’ ideas** | Workshop rules | Refer to the large printed: **“YOUR ROLE, YOUR NEEDS”**   - Everyone’s opinions are valid - We ask that you only focus on YOUR ROLE and YOUR NEEDS as a patient/clinician – try not to assume what the other patient/clinician believes or is trying to do, this is why we invited both - Everyone should feel comfortable and not judged: - everyone wears different hats, experiences situations differently and we need to respect their opinions but feel free to ask clarifying questions or respectfully disagree - Enthusiasm is great but gentle reminders will be given if we get off track - Sessions will be tape recorded but it can be turned off upon request - Everything in the room will be confidential – no names please - No identifiable information will be recorded - Honest feedback is best to improve research and the system – no need to “save face” - No treatment advice will be discussed during this session - If questions of medical issues arise, please note them and discuss later with your own clinician |
| **A: Increase engagement/participation**  **P: Reassure that all ideas have value and no idea is stupid, breaking the tension and making the atmosphere more casual** | Warm up activity: Worst Possible Idea exercise | ***Practical notes:*** *White pieces of paper to draw on or write ideas on (enough for all 7 participants, researchers included)*   - **Purpose** is to reinforce that no such thing as a bad idea because everything leads to something useful: umbrella - **Rules:**   - - Both research team and patients will participate     - Everyone should get a chance to talk, but okay if there are no ideas - **“Worst Possible Idea” exercise^[[1]](#endnote-1)^**   - - Think of the worst design for an umbrella     - Write down as many ideas as you can on separate white sheet in 2 minutes     - Pass them to the person next to you and elaborate on their bad idea – go through as many separate ideas as possible for 2 minutes X 2 rounds     - Then read out loud and place them on a board visible to all     - Now propose the best design – no limits (gravity, legal issues, reality etc.)     - Plan B: MB comes up with an example in Norwegian |
| **Break (9:40-9:50)** | | |
|  | Introduce main workshop activity | - Start audio recording of the session - **Explanation:** we will brainstorm and write down some things on our own and then discuss together for most of today – this way, in case we are not able to cover everything, you are still able to provide your input! - **Introduce the landscape**: Explain what the landscape is and how we will use it during the day   - - Fargelegg Landskapet: Egen behandling, Møte, klinisk diabetespraktis (MB put up graphics to illustrate this) After each question you will be given a few minutes to brainstorm - This is just brainstorming, you can keep writing down ideas as we go so don’t think that 2mins for brainstorming - Feel free to post it yourself or hold it up and we will come get it from you |
| **A: Which parameters patients gather, their baseline motivation level and measure of activation on a non-standardized level**  **P: Understanding what patients prioritize in their self-management, which will primarily inform the functionalities of the system and secondarily enable clinicians to be aware of patients’ perspectives** | **Discussion:** Do you feel like you self-manage your diabetes? Why do you self-manage the way that you do? | ***Practical notes:*** *Audio only + cotton balls to initiate conversation and set precedent for equal participation*   - **Cotton ball method:** each person gets 3 and “uses one up” each time they speak. - **Follow-up & examples to use if the conversation stalls**    - - why do you choose to spend more time on one thing than another     - Impacts of the disease?     - Realizations, habit changes?     - Confidence and self-efficacy/knowledge of disease?     - Challenges?     - Motivations?     - Overall perceptions of how well they self-manage? |
|  | - - - 1. What do you focus on? Do you have a **goal** for any of these or do you just track to find trends or patterns?       2. What data are you saving/collecting? | ***Practical notes:*** *Yellow post-its to write or draw on in response to each question (1-2minutes per question)-* ***1 verb and 1 noun***   - **Instructions:** mark each post-it as # to reference the question we are on and try to use only **1 verb and 1 noun for each feedback** - **Follow-up questions & examples to use if conversation stalls**   - - Are you gathering: BG, physical activity, diet, medication?     - Routines, following a schedule, preparations? - Place responses on white board or wall under heading **“Egen behandling”** |
| **A: What tools and support patients use to aid their self-management**  **P: Depicts possible areas besides health measures that the system can/should address** | - - - 1. Do you use any mHealth apps or medical tools and which kind? And what is good and/or bad about them? (else it will just be a survey) | ***Practical notes:*** *Yellow post-its to write or draw on in response to each question (1-2minutes per question)*   - **Instructions:** mark each post-it as # to reference the question we are on and try to use only **1 verb and 1 noun** - **Follow-up questions & inspirational ideas**   - - Smartphone app, smartwatches, BG meters - Place responses on white board or wall under heading **“Egen behandling”** |
|  | Participants place post-its on board in area drawn | MB reads out loud to begin discussion and allow people to comment on the ideas “hva synes du om dette forslag…””.. ” |
|  | **Discussion:** How do you use **these tools** to help you? | ***Practical notes:*** *Audio recorded only, no additional supplies or preparation needed*   - **Follow-up questions**   - - why are they effective or why not?     - how could they be more effective…”its good but” |
|  | - - - 1. What challenges do you have with self-management?       2. How do you (or could you) overcome that?   **Discussion question:** ask them to comment on these suggestions – lets focus on the solutions or any other ideas given other tools etc. that could help solve the challenges? | ***Practical notes:*** *Yellow post-its to write or draw on in response to each question (1-2minutes per question)-* ***1 verb and 1 noun***   - **Instructions:** mark each post-it as # to reference the question we are on and try to use only **1 verb and 1 noun.** Post suggestions for 4 and 5 next to each other - Place responses on white board or wall under heading under **“Egen behandling”** |
| **A: Determine how patient participants interpret their responses.**  **P: To engage participants throughout the activities. Allow them to take ownership of what is produced.** | Creating topic headings together | ***Practical notes:*** *MB at board to organize*   - **Instructions:** now that we have some ideas on the board, we should organize them so that it will be easier to reference these in the coming activities (because we will be using your suggestions in the future) - **Follow-up questions & examples to use if conversation stalls**   - - Habits? Tools? Technology? Data? Goals? |
| **Break (10:40-10:50)** | **Break (10:40-10:50)** | |
| **A: Which data displays are easiest for patients to relate to. Baseline understanding and engagement in diabetes data and disease knowledge.**  **P: Understanding how patients interact with their registered data, thereby setting a basic or baseline way of displaying data in the system** | - - - 1. How do you use your data to self-manage | ***Practical notes:*** *Audio only*   - **Instructions**: looking at what we wrote for questions 1&2, let us discuss this. - **Plan B: Give example situation to respond to** - **Follow-up questions & examples to use if conversation stalls**   - - How would you use your data to make a change or decision in self-management? |
| **A: Which data do patients want to share and what answers do they need from their clinicians to understand their data and act on it in self-management**  **P: The expected scope of the systems functionalities that will facilitate discussion, initiated by patients** | - - - 1. What are clinicians helping with?       2. What could they help with?       3. What should they help with?   **Discussion:** Have you presented your own data or app data to your clinician? When did you or think that you should present your data? What worked/was successful or easy and what didn’t work or you both had trouble discussing? | ***Practical notes:*** *Yellow post-its to write or draw on in response to each question (1-2minutes per question)-* ***1 verb and 1 noun***   - **Instructions:** mark each post-it as # to reference the question we are on and try to use only **1 verb and 1 noun.** - Place responses on white board or wall under heading **“Egen behandling”** |
| **A: Generate ideas to facilitate joint discussion**  **P: Prepare participants for joint session – thereby saving time and establishing concrete topics to discuss** | **Prepare for lunch break:**  Given this new situation where you gather data yourself…   - - - 1. How do you think this changes yours and your clinician’s roles during the consultation?       2. Does it change what you bring to the consultation or how you prepare to talk to your clinician? | ***Practical notes:*** *Brainstorm-write down or draw (if they want) ideas on post-its (can take them during lunch)*   - **Instructions:** because there is no such thing as a free lunch, we would like you to think through these questions and jot some things down because we plan to come back and discuss consultation during the joint session. Place responses on white board on line between **“Egen behandling”** and **“Møte” -** when you return from lunch |

| **(Stop audio recorders) Patient Lunch (12:00-13:00)** | | |
| --- | --- | --- |
|  | patient participants to lunch in cantina at 12:00 | - - *Preparation tasks for research team* - *Workshop rules Bring lunch in the room for the clinicians and 3 researchers - eat when we prep them and MB and AG can help describe the purpose and project etc.* |
| **(Start audio recorders) Clinician Session (12-12:55)** | | |
|  | Introductions of participants | - **Welcome and introduction:** First Names and Experience with diabetes patients |
| **A: Common understanding of what we want to achieve from workshop**  **P: Working within the scope of the project without limiting creativity and input** | Description of project purpose and aim  Overview of what was done in the patient session | - - Current situation of mHealth and EHR data - Purpose of the day and project – making the most out of this situation - Introduce the Co-design concept (do this also for patients) - Make clear what the patients have done before they arrive and what kind of input we want in this workshop - Make clear what will help a safe environment – no judgement and no assumptions of how patients perceive and think about their diabetes - Generally about the FullFlow and actors by EÅ - **Ask if they are comfortable with MB and AG introduce Full Flow project in English:** - **MB and AG**, 2-3 sentences about purpose of Full Flow and the fact (no details) that we are PhD students, and what they want feedback on. - Visually present the ideal situation that we hope to achieve   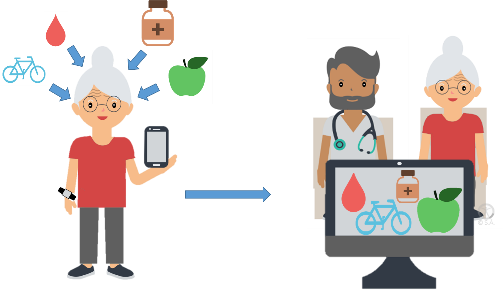   - - **(Refund formalities – if not already done by AsGr)**   - **Opportunities for future involvement** - This is a large project and we hope to include you in the future if you are interested – what would we need in order to recruit? And approximate interest from these participants? - **Purpose of this workshop** is to generate concrete suggestions using post-its AND fruitful and concrete suggestions and feedback during discussions that we can use to design this ideal situation *(above)* - **Explain the landscape poster:** This is what was produced during the patient session, i.e the yellow post-its on the boards. We will be doing the same thing in this session. Please keep in mind that our aim is to cover this whole landscape today together |
| **A: Common understanding of how to conduct one’s self during the workshop**  **P: Establishing a safe and comfortable environment for ALL participants to share own ideas and receive others’ ideas** | Basic workshop rules plus clinician session-specific rules | Refer to poster on wall that says **“YOUR ROLE, YOUR NEEDS”**   - Everyone’s opinions are valid - We ask that you only focus on YOUR ROLE and YOUR NEEDS as a patient/clinician – try not to assume what the other patient/clinician believes or is trying to do, this is why we invited both - Everyone should feel comfortable and not judged: - everyone wears different hats, experiences situations differently and we need to respect their opinions but feel free to ask clarifying questions or respectfully disagree - Enthusiasm is great but gentle reminders will be given if we get off track - Sessions will be tape recorded but it can be turned off upon request - Everything in the room will be confidential – no names please - No identifiable information will be recorded - Honest feedback is best to improve research and the system – no need to “save face” - No treatment advice will be discussed during this session   **Clinician session specific rules**   - - During this session, feel free to speak about your perceptions of patients, but during discussions with patients, we ask that you only focus on YOUR ROLE and YOUR NEEDS     - E.g. of acceptable phrasing: “ideally it would be good to get X amount of information from the patient”- objective and general in comments   - Suggest functionalities that might be used in a good way – do not focus on barriers, focus on what you need and how you would do it regardless of how much time it takes because the technology is intended to help this |
| **A: Baseline of how clinicians conduct their consultations and interact with patients**  **P: Same as previous** | 1. How do you start the consultation? (as separate Post-it sessions?) 2. What do you cover in a typical consultation?   **Discussion:** What information (either from memory, written or apps) that a patient presents does and does not have an impact on decision-making? Examples please.  **Discussion:** How can patients’ use of mHealth and patient-gathered data be beneficial in the consultation? | ***Practical notes:*** *Green post-its to write or draw on in response to each question (1-2minutes per question)-* ***1 verb and 1 noun***   - - **Instructions:** mark each post-it as # to reference the question we are on and try to use only **1 verb and 1 noun.**   - **Place responses** on white board or wall under heading **“Klinisk praksis”** |
| **A: When clinicians believe shared decision making is appropriate.**  **P: Brainstorm situations in which the Full Flow might be more useful for GPs and T2Ds** | 1. **When** would data-sharing be useful or appropriate, i.e. in which situations?   **Discussion:** Have your patients ever brought you data on or from an mHealth app? Do you trust it, what are Benefits? Challenges? Opportunities?  *(don’t want written for patients to see)* | ***Practical notes:*** *Green post-its to write or draw on in response to each question (1-2minutes per question)-* ***1 verb and 1 noun***   - - **Instructions:** mark each post-it as # to reference the question we are on and try to use only **1 verb and 1 noun.** Your responses can be theoretical or reality that they have experienced. - **Place responses** on white board or wall under heading **“Clinisk praktis”** - **Follow-up questions & examples to use if conversation stalls**   - - If patients have questions, if you gave them a specific task? |
| **(Stop audio recorders) Break (12:55-13:00)** | | |

| **(start audio recorder) Joint Session (13:00-15:00)** | | |
| --- | --- | --- |
| **A: Common understanding of how to conduct one’s self during the workshop**  **P: Establishing a safe and comfortable environment for ALL participants, establishing that all input is equal and valued** | Ice breaker activity: Year of the Coin | Different coins with different years than the morning session   - - Something that happened in your life during this year |
|  | Reiterate workshop rules | Refer to poster on wall that says **“YOUR ROLE, YOUR NEEDS”**   - Everyone’s opinions are valid - We ask that you only focus on YOUR ROLE and YOUR NEEDS as a patient/clinician – try not to assume what the other patient/clinician believes or is trying to do, this is why we invited both - Everyone should feel comfortable and not judged: - everyone wears different hats, experiences situations differently and we need to respect their opinions but feel free to ask clarifying questions or respectfully disagree - Enthusiasm is great but gentle reminders will be given if we get off track - Sessions will be tape recorded but it can be turned off upon request - Everything in the room will be confidential – no names please - No identifiable information will be recorded - Honest feedback is best to improve research and the system – no need to “save face” - No treatment advice will be discussed during this session |
| **A: Presentation and common understanding of the “resources”, i.e. common tools and ideas, that we can use or refer to during the discussion.**  **P: Ensure that ALL participants are aware of what we have to work with and each other’s ideas.** Activity will limit group think and allowing all to contribute even if they are not comfortable being vocal. | Present where we are in the landscape: | - **Introductions to the joint session:** This is what we have produced together so far today and we can use these during the discussion if they are useful   - - - - The morning session produced these – briefly review     - - the lunch session produced these- briefly review   - **The overall goal is:** to discuss together, how to meet both of your needs that you have brainstormed during your separate sessions, to be used during the consultation*.* We want to produce concrete suggestions on post-its AND fruitful and concrete suggestions and feedback during discussions  - Allow time to ask questions and discuss or clarify these before beginning the activity |
|  | Participant review of the landscape themselves | - - **Instructions:** now we should take a few minutes to all of us go up and physically look at what we have produced together – both your own and the other part. Please tell us what you agree with (endorse the idea) by placing a *check mark* (or red page marker) on the post-its that you truly agree with and you think should be highlighted in the coming discussion Each get 3 pieces to place out. |
|  | **Break (14:00-14:10)** | |
|  | Describe activity and then Present the Data Sharing Story Board | - - **Instructions:** Our intention is to colour in this landscape by filling in the important components/aspects of the situation whereby we can all discuss and agree on what is important for data-sharing. Once each person has described “why or why not” during the post-it phase, then we can open up and ask “who had similar suggestions, any comments? Arguments?” |
|  | Describe how the activity will be conducted | - - Everyone should write down on post-its their ideas before presenting them out loud/discussing so that everyone has a chance to contribute and discuss. After discussion, the post-its are placed into the landscape   - **Cotton Ball method:** each person gets 3 and “uses one up” each time they speak. |
| **A: When/how/what mHealth data should be shared?**  **P: To establish a foundation for the discussion which will direct how and when the Full Flow system should be used, which will inform which functions are appropriate for each use case.** | - - - 1. **When** would it be appropriate to share self-gathered information and data during consultations between patients and clinicians? **(more than today)** | ***Practical notes:*** *Respective post-its to write or draw on in response to each question (3minutes per question)-* ***1 verb and 1 noun***   - **Instructions:** mark each post-it as # to reference the question we are on and try to use only **1 verb and 1 noun.** Each person will be allowed to read theirs out loud and explain their perception. **Please** be prepared to say why or why not they wrote that on the post-it during the discussion - **Discussion & Cotton ball exercise:**    - the point of this exercise is to allow everyone a chance to speak and then we can open it up once all cotton balls are used up or everyone speaks that wants to.   - is anyone willing to say why or why not there is an appropriate time to share self-gathered info/data?   - Does anyone have any additions or comments to this? - **Follow-up: (**Once cotton balls are all used)   - E.g. to use if silence: When the patient has specific questions?   - When there is sufficient data?   - During the planning phase during diagnosis and then agreed upon thereafter for specific changes in self-management?   - Why is the suggestion effective? - **Place responses** on white board or wall under heading **“When” under “Møte”**, once the discussion has come to an end or we have reached the time limit then everyone should place their post-its. |
|  | - - - 1. **What** self-gathered information and data would be useful to share during consultations? *(refer to examples that both brainstormed in separate sessions: suggestions for self-management were XYZ and suggestions for what clinicians need to know for their clinical practice were ABC- MB can present some of these)* | ***Practical notes:*** *Respective post-its to write or draw on in response to each question (3minutes per question)-* ***1 verb and 1 noun***   - **Instructions:** mark each post-it as # to reference the question we are on and try to use only **1 verb and 1 noun.** Each person will be allowed to read theirs out loud and explain their perception. **Please** be prepared to say why or why not they wrote that on the post-it during the discussion - **Discussion:** is anyone willing to say why or why not these data should be shared during the consultation?   - Does anyone have any additions or comments to this? - **Follow-up:** How much information should be shared? Very details or overview? 🡪 What should not be shared or it is not necessary to share during consultations? |
|  | - - - 1. **How** can each of you use this information to a) either make a joint decision about self-management or treatment or b) make own decisions about how you will use this information in self-management or your clinical practice? | ***Practical notes:*** *Respective post-its to write or draw on in response to each question (3minutes per question)-* ***1 verb and 1 noun***   - **Instructions:** mark each post-it as # to reference the question we are on and try to use only **1 verb and 1 noun.** Each person will be allowed to read theirs out loud and explain their perception. **Please** be prepared to say why or why not they wrote that on the post-it during the discussion - **Discussion:** is anyone willing to say why or why not these data should be shared during the consultation?   1. Does anyone have any additions or comments to this?   **Follow-up:** |
|  | Refer back to main idea of the project: data sharing system  Then ask participants: do you have any good ideas about how to visualize your data?   - Draw on a blank paper | - 1. Now we have gone through what you need and what questions and purpose you have for the consultation. With these ideas in mind, we want to now figure out HOW a system should work and what pictures or graphs or information the system should have that visual triggers the discussion of what you need to know   2. Present the wireframes of graphs and plots that they can draw on if they want AND graphics for inspiration   3. One white sheet and one with some wire frames on it      1. *MB and AG: print out several copies pictures and descriptions of these that they can draw on or pull out to refer to*   Allow for some time to ask questions and clarify or give initial thoughts   - **Prompt them to draw ideas:** Before we present our sketches and ideas, we want to give opportunity to brainstorm how to go about sharing data and how it should look   - *Reinforce that it is okay if no one has any ideas, this is what the next activity is for* |
|  | Briefly present the concept of the data sharing system in a bit more detail than previous sessions: *explain* Introduce research team’s ideas for core components of the system and rough sketch:   - Use all ideas provided to suggest good views/presentations. | ***Practical notes:*** *MB to put three scenarios, one for each situation on the landscape in the middle area (MB to print the scenarios – computer screens with headings)*   - **Introduction of researcher ideas:** To be more concrete, here are some things that we have played with and discussed amongst ourselves (MB to put up wire frames and graphics etc. - **Explain the purpose of the system in more detail:** the stepwise treatment approach/the fact that the system should progress or evolve with the patient and disease, e.g. no data and just goals to graphs and calculations)   1. **Present wireframe components ALONG WITH:** pictures AND their own graphs that they can now place in the scenarios.:   - Wire frames   - Goal setting and progress tracking   - Presenting only the data that patient gathers and decides to share   - Options for what data types are possible to collect during the study   - Ability to ID trends and possible problem areas - **Three situations:** Imagine that this is implemented already- how should it look like when….   - Shortly after diagnosis   - Learning to self-manage and understand disease   - Today   Describe that these are example scenarios to illustrate the progress of the system in different situations |
| **A: How should:**   1. **the system look (i.e. level of detail and functionalities) at each individual stage of the disease and patient’s self-management** 2. **data be displayed** 3. **the system facilitate follow-up from one consultation to the next (facilitate continued care)**   **P: How the system can display information in a mutually understandable way that also facilitates continued and collaborative care** | 1. 5.a. Consultation in the beginning of treatment/ shortly after diagnosis 2. 5.b. Consultation during your “learning period” – months or years into self-managing the disease | - **Instructions:** Now we will present the scenario from the two perspectives and give you some time to brainstorm, just as before, some notes and ideas about how this should look. Please write down or draw your ideas and then we will post them on the board and discuss together. - **Scenario 1 & 2 Consultation shortly after diagnosis and in the middle of disease management:** you first hear that you have diabetes and clinicians, you have given them information to review on their own. Imagine that you both have had previous meetings that go over some basic information and. Now, you are both back to continue planning treatment and discussing the future. - **Follow-up questions to patients:** Thinking back to the first months or years of your diagnosis. Knowing what you know now about self-managing, what works for you and support you need –   - What should the system display as options for the consultation discussion – meaning you have gathered some data, how do you want to show it so that it makes sense for you?   - what kinds of images and what should they include that could be helpful to you?   - What other information should the system provide to help plan your treatment? E.g. goals for how to start self-management or data gathering options so that you can select where to start? - Allow participants to write down additional thoughts on post its, wireframes (either drawn on or not) before proceeding *– 5 minutes to brainstorm* - **Follow-up questions to clinicians:**    - what do you look for in the small/large amount of data that you have instructed your patients to gather?   - What helps you determine the progress that your patients are making?   - What problems or indicators of challenges might you look for? - Allow participants to write down additional thoughts on post its, wireframes (either drawn on or not) before proceeding *– 5 minutes to brainstorm* - Referring to the story board, have participants place the post-its on the board in the appropriate location – *3 minutes* - Ask if anyone would like to describe their placement of the ideas - Researcher can say aloud what seems to be important for the system from patients’ and clinicians’ via the post-it notes - Allow participants to discuss what is shown before moving to next situation – *15mins* |
|  | **Break (as needed)** | |
|  | 1. 5.c. Today’s consultation | - **Scenario 2 Today’s consultation:** Think of the last consultation that you had as an individual – you have been working with your diabetes for some time and have learned more about yourself and what helps you manage your health and how to interpret your data. And clinicians: you are now meeting with a patient who has had the disease for a while and has been collecting data. - **Follow-up questions to patients:**    - - - - what data did you bring? What questions did you have for your doctor?         - If the system were there to display your data, what kinds of graphs or progress or activities would it show? What should the graphs indicate (e.g. highs and lows of BG, each individual registration or summaries of your data, calculations of X? (E.g. Single events, daily and weekly trends)         - Describe how you would show this to your clinician? Where do you start? Does anything need to change? - **Follow-up questions to clinicians:**    - - - - If you were to use this system, ideally, how much data do you need to understand your patient’s situation and suggest improvements or explanations?         - What should the graphs or figures show – how much detail do you need and what kind of detail, e.g. averages, calculations, medication adherence, indicators of change in their habits/goals or health progress?) - Allow participants to write down additional thoughts on post its, wireframes (either drawn on or not) before proceeding *– 5 minutes to brainstorm* - Referring to the story board, have participants place the post-its on the board in the appropriate location – *3 minutes* - Ask if anyone would like to describe their placement of the ideas - Researcher can say aloud what seems to be important for the system from patients’ and clinicians’ via the post-it notes   Allow participants to discuss what is shown before moving to next situation – *15mins* |
| **Summing up and closing remarks (15:00-15:15)** | | |
| **A: Common understanding of what was covered, how research team will incorporate these ideas into the next stages and future steps of the project**  **P: Inform participants that their input was valued. Also that there are future opportunities to participate.** | Sum-up what we have all done together and our plans for future activities etc. | - Reiterate the purpose of the project and why this research is important - Researchers emphasize their thanks to all participants - Have a description ready for exactly how this will inform the study and how we will test it out during the Run-In period - Tell them that we will send out additional information for future study opportunities WITH summary and follow up questionnaire about their experience with the co-design and any other ideas they may have (2-3 weeks after) - Provide research team’s contact information (visit cards) |

1. <https://www.interaction-design.org/literature/article/learn-how-to-use-the-best-ideation-methods-worst-possible-idea> [↑](#endnote-ref-1)
