# Supplementary material for: How mHealth can facilitate collaboration in diabetes care: qualitative analysis of co-design workshops
Source: BMC Health Serv Res. 2020 Nov 30;20:1104. doi: 10.1186/s12913-020-05955-3 (PMC7706243; doi:10.1186/s12913-020-05955-3)
Supplement: Supplementary file 2 — Additional file 2. [file 12913_2020_5955_MOESM2_ESM.docx]

# Additional File 2. Additional information and quotations extracted from the co-design workshop transcripts

We believe that context is of equal importance to design features when understanding how mHealth can impact end-users. The following additional information provides a foundation of understanding of priorities, experiences, challenges and wishes individuals with diabetes have for themselves and from their healthcare providers about diabetes care. In doing so, this information contextualizes and provides background for the input gathered about experiences, expectations, preferences and concerns about using patient-gathered data during diabetes consultations. This feedback helps us – as researchers, healthcare providers and health authorities – understand if and how mHealth technologies and sharing patient-gathered data can address these factors and facilitate a beneficial change for both end-users, i.e. patients and healthcare providers. The following additional quotations and description of the discussion (i.e. summary) are separated into the identified themes and sub-themes presented in the main manuscript. The codes provided are those that were identified in the original qualitative analysis.

#### Theme 1: Patients’ and providers’ need for more specific and detailed information in diabetes care

**Table 1.** Additional quotations, grouped by codes assigned during qualitative analysis, from participants with T2D about their diabetes self-management

| **Code** | **Summary** | **Quotation** |
| --- | --- | --- |
| Self-management aim | - Welling - Accept responsibility | *“Wholeness and well-being is the focus. The goal is well-being” (T2D_P2)*  *“Pretty much it is ourselves that need to find out about things” (T2D_P1)* |
| Self-management habits | - Focus on diet, exercise - Diabetes-specific info sources | *“Diet and physical training or exercise – at least at my age exercise is the most important in addition to diet. The goal is to stay as low [blood glucose] as possible, maybe satisfy the doctor’s wish” (T2D_P3)*  *“I use internet and read about diabetes. Every time I eat something new, I can search it up. See how the specific food is built and contains, and if there are any objections about it” (T2D_P3)* |
| Motivation | - Symptoms as external motivators - Peer support | *“Abnormal warm and sweating and thirsty, then I measure blood glucose extra, to see my value. If I am tired, I also measure, and normally have low blood glucose. Have to add sugar of some sort, juice is the best. The excuse being you can eat chocolate if you want. I use my feelings, then measure, then take action” (T2D_P3)*  *“I am in a work out group circle, once a week….If not for that I would give up the diabetes battle, considering I feel like go to the doctor every week and get feedback about blood sugar. It always varies. So basically you rely more on physiology than on your own ability to control it” (T2D_P3)* |
| Challenges | Diabetes-specific info sources | *“I try to read online magazines, but there are so many articles contradicting each other” (T2D_P4)* |

**Table 2.** Additional quotations, grouped by codes assigned during qualitative analysis, from participants with T1D or T2D about their experiences with diabetes healthcare providers

| **Group** | **Code** | **Summary** | **Quotation** |
| --- | --- | --- | --- |
| **Participants with T1D** | Frustrations with healthcare services | - Experienced less support than had hoped for - Unsure about whether to contact HCP or not | *“I suppose I can call my diabetes nurse but she has never called the healthcare service in the 30 years I have had diabetes to solve a situation today. I have called the diabetes line a few times but they are too far away the specific situation, so they tell me to go further to someone else and then you get answer after a day or two, but that is not when I am in the situation. So being able to solve the situation that you are in… I don’t want to disturb doctors and nurses with my small problems. But they are maybe not so small if we… acknowledge what they really are” (T1D_P3)* |
| **Participants with T2D** | Positive experience with healthcare services | - Rely on healthcare providers to tell them the status of their health - Experience discussion with GP about patient-gathered data | *“I go to yearly controls and take many blood tests. If I don’t hear anything, I assume everything is fine” (T2D_P2)*  *“My GP helps with control and coping and looks through the Diabetes Diary [app] on my phone…and then we start discussing” (T2D_P1)* |
|  | Frustrations with healthcare services | - Experienced lack of training/ support/ knowledge - Experienced little use of healthcare services - Limited contact with HCPs | *“The experience I have with the doctor’s office is that GP usually is not there, so always a substitute, which it has been the last three years….I feel like my general practitioner doctor that they really lack the knowledge in which we diabetics struggle with. Patients are just a case, do not have enough education to cope with that part of public health” (T2D_P2)*  *“After I was diagnosed with diabetes, I haven’t gotten any advice… I got an appointment at a nurse. I got a note on how to react on insulin and increase doses eventually. [I was trained] to use the syringe. And three months after to check, and then he was satisfied with HbA1c. After that, I haven’t talked to anyone about diabetes at all, regarding healthcare professionals” (T2D_P2)* |
|  | Wishes for healthcare services | Desire more guidance | *“I have little contact with healthcare professionals in relation to diabetes. I was diagnosed three years ago. Was two-three times at my GP. After that, I have felt like I had enough control myself for it not being a necessity. But now I feel like I can use some more contact and input. So a question being if an app which gave more feedback rather than just the average numbers, would be a possibility” (T2D_P3)* |
|  | Understanding of healthcare services | Understand HCP’s needs and limitations | *“I don’t think GPs have the time it requires to get familiar with it…. It’s not so easy you know; the GP have 1000-2000 patients” (T2D_P3)* |

**Table 3.** Additional quotations, grouped by codes assigned during qualitative analysis, from healthcare providers’ experiences with patients with diabetes

| **Group** | **Code** | **Summary** | **Quotations** |
| --- | --- | --- | --- |
| **Specialists** | mHealth (positive) | - Positive toward mHealth - mHealth can be easy to relate to - Experience mostly with medical devices (CGMs) - Use of mHealth with EHR systems | *“We are very positive about [mHealth and diabetes care], because we have been in the field for a long time. And we see that it’s a revolution. It’s fantastic. The fall of late complications in enormous” (Specialist1)*  *“Some of it is very easy - in using the DIPS journal system or NOKLUS [the national diabetes registry]. Ideally when a patient comes to me, I can automatically - by Bluetooth or something - get the continuous glucose values for the last week into my electronic diabetes journal system, with insulin taken and glucose measured, so I could have it on the screen and then I could have a look at it” (Specialist1)* |
|  | mHealth (negative) | - mHealth is a challenge to clinical practice capacity - HCPs do not have time to learn the new technologies | *“In our business where we are only measured after how many patients we see every day. So if we go to meetings and learning things to learn to use technology it’s on the minus side because we do not see patients” (Specialist1)* |
|  | mHealth (neutral) | - Learning about mHealth: literature - Experience with mHealth as learning strategy - Prefer learning by experience than by industry “sales pitches” | *“When we are talking about new technology, it’s mainly based on CGM. Because that’s the new technology the past 10 years” (Specialist 1)*  *“[Instead], we generally read scientific literature…and experience from all of my diabetes nurses and sort of accumulating clinical experience from the use of these devices… which means we see the problems much better than getting the industry coming into the door and telling about “look at the new device” (Specialist1)* |
|  | Priorities of clinical practice | - Coordinate resources - Achieve the greatest good for the most people - Provide support for those who need it | *“CGM is very costly, not only to buy it for... but also costly because it uses all of the time resources for our diabetes nurses” (Specialist1)*  *“As to resources and as to budget costs, it is much more helpful or much more saving money and saving pain for people to bring these people here with HbA1c of 10% down to HbA1c of 8%. That is really an advantage for everybody” (Specialist2)* |
|  | Perception of patients | - Perception of patients’ concerns and motivations - Patients’ locus of control in diabetes - Some do not communicate all symptoms to avoid being hospitalized | *“Of course we want that to be more around 7% but many patients don’t want to go that low because they fear hypoglycaemia and they think it would cost too much work and they don’t understand why they should be lower, because they think the late complications are far away” (Specialist2)*  *“I think the main responsibility is what [Specialist 2] says, is to make them communicate with us because that’s a big problem. That patients don’t want to come and communicate with us and they find it bothersome so they go to their general practitioner and get an insulin prescription once a year [to avoid hospital admissions]” (Specialist1)*  *“This is in general about health and psychology like locus of control -. Is the locus of control of my health within in myself or am I just behaving healthy because you and you and you tell me to – that’s a general problem that is exaggerated in diabetes” (Specialist1)* |
| **GPs** | Experience during diabetes consultations | Typical consultation experience with someone with T2D | *“Many diabetes controlled are mixed with a lot of other things and I feel that can be confusing because high blood pressure, maybe overweight, maybe low back pain, maybe a lot of other things. I measure the blood pressure, I listen to the heart and lungs, we talk, if they have any problems with the medic since diabetes medicines often has side effects, so we discuss that” (GP3)* |
|  | mHealth (positive) | mHealth technologies as motivation | *“When I think of some of my patients, [I see that] they are so fed up with diabetes” (GP3) [and mHealth could help with motivation]* |

#### Theme 2: mHealth technologies’ impacts on patients and providers

##### Subtheme 2A: Purposes of, and challenges related to, mHealth and patient-gathered data

**Table 4.** Additional quotations, grouped by code, from participants’ experience with mHealth and/or patient-gathered data

| **Group** | **Code** | **Summary** | **Quotations** |
| --- | --- | --- | --- |
| **Participants with T1D** | Experience with mHealth data | - Use of mHealth helps with/ motivates self-management - Use of multiple devices - Reliance on app for self-management - Apps allow one to reflect on previous self-management experiences | *“Data collection gives you experience…if you track the data” (T1D_P3)’*  *“When I have it on my phone it is easier to plot insulin and have data on a specific time, amount insulin, blood glucose, and maybe food, if I bothered to” (T1D_P4)*  *“I registered them in Diabetes Diary app, but there I also note the physical activity. I tried registering in the new version my medicine, but I gave up because I had to keep up 13 different tables every day. …also I use, and another app [step counter] from Samsung where it was nagging that I am not physical active enough in day time…. If you try to be independent of your mobile phone, you won’t be able to follow anything. (T2D_P3).*  *“[I collect] a lot of blood glucose measures…too much…I only measure in the morning to see the value [which] affects how I respond”, he reflected on longer-term effects like “the results for stress level, drinks and such” (T2D_P1).* |
|  | Desires for mHealth | - Change: additional interoperability - Future use: collecting data to present to HCP | *“To start, I would want that my measurements and phone should talk together….if I don’t bother [to collect data consistently] it would be nice for a period – a week- before I have a consultation. Blood glucose for several months is available but food and insulin I take for a set period can be gathered. I don’t picture me register all of insulin and all food I eat, but for a period would be nice.” (T1D_P4)* |
| **Specialists** | Perceptions of mHealth | - Patients do not want data-overload - Automatic data-gathering is necessary | *“So you need to automate things because patients cant bother or are not happy about using all of these data” (Specialist1)* |
|  | Perceptions of patients and mHealth | - Who uses mHealth - CGMs perceived as mHealth - Patients gather a lot of data but do not always reflect on it | *“I think some of them are younger or older, the people in the middle don’t have time for all of this. Because they are early in their careers and they make a family and they have children so they have got time for all of this and then if they the CGM people –they can come with all sorts of data because its automatic but they haven’t made a diary or sort of explained why was it like this, why did I get a hypoglycemia” (Specialist1)* |
| **Participants with T2D** | Use of mHealth in consultations | - How mHealth could change diabetes consultations - Expectations of GPs related to mHealth | *“It ends up with the doctor often being a conversationalist. Of course they want to help you but I experience that they don’t have time” (T2D_P2).* When asked by the researcher facilitating the discussion if they thought sharing their patient-gathered data would change this, the participant responded*, “I don’t know, could have compared data, and told me what I should do. As you say, maybe not the GP should be the one, but a different healthcare professional. Maybe expect too much from the GP” (T2D_P2)* |
| **GPs** | Experience with patient-gathered data in clinical practice | - Reliance on data from patients for information - Observation of which data-types patients collect/bring - Reliability is important | *“If they bring their books, and they’re clever sometimes they just test three days before and then stop testing for half a year, and then come back with three lost test days. Some are testing every day, four times a day…Some have blood pressure machine at home, then they show me…. I rely on my patients [for information]” (GP3)*  *“Glucose meter yes. Usually bring it yes. And the books, usually blood sugar values, some people bring blood pressure tests” (GP1)*  *“If they have some information that we can rely on, a book, writing their values, few of them is quite clear to remember. If they have reliable information, we use that more than medical history because things happen on the way” (GP1)* |

#### Theme 3: Data-sharing system

##### Subtheme 3A: Expectations of sharing and receiving PGD during consultations

**Table 5.** Additional quotations, grouped by code, from participants’ about their experiences and expectations about sharing and receiving patient-gathered data during diabetes consultations

| **Group** | **Code** | **Summary** | **Quotations** |
| --- | --- | --- | --- |
| **Participants with T1D** | Experience using mHealth with HCPs | - HCPs understand patients’ situation - Positive experience discussing mHealth data with HCP | *“I have good help in my diabetes nurse because I can talk with him [about own-gathered data]… because it’s no one else I can talk to who understands” (T1D_P4)* |
|  | Expectations for sharing patient-gathered data | - More support - Wish for HCP to see data during consultations | *“I wish that [HCPs] could see it when I’m at checkups….maybe [HCPs] can help me more if they see that there’s a reoccurring problem…if I’m high during the evening...we can try to talk more specifically” (T1D_P4)* |
| **Specialists** | Negative consequences of mHealth | Consequences if patient is not prepared to explain own-gathered data | *“[The doctor] is going to be the person who is solving all the patient’s problems” (Specialist2)*  *If the patient has not reviewed or attempted to understand their own-gathered data before sharing it with the doctor, “then it’s useless” (Specialsit2)* |
|  | Recommending medical devices | - Technology not appropriate for everyone - Support technology use for specific groups of patients | *“For some individuals, I put a [continuous glucose monitor] on them and say “you are supposed to use it” while others, I keep it away from them” (Specialist2)*  *“We also have a lot of compliant patients who try their best. So those in the group below or around 7% are compliant and they meet for consultations and want the new insulins and they want the new technologies – so that is no problem” (Specialsit2)* |
| **Participants with T2D** | Expectations for sharing patient-gathered data | Better communication | *“Better communication about your situation if you have your data and can evaluate from it…. As I see the GP, you go to them when you have a specific problem. If you have control with diabetes, it is not a specific problem” (T2D_P2)* |
| **GPs** | Expectations of receiving patient-gathered data | - GPs should not have to use new technology if patient comes prepared | *“If you need the GP to go to seminars to learn about it they will do it- they may not necessarily download it….If the patient comes with [PGD] and it is easy to understand” (GP2)* |
|  | Expectations of discussing patient-gathered data | - Patient-gathered data could help streamline discussion - Patient-gathered data could provide more specifics and evidence of patients’ self-management | *“The discussion with the patient could at least be more to the point. Now we ask “Have you been exercising lately?”, and they say “Yes, I have been every day”, and then “Okay”. Instead – “I see you have not been exercising every day or at all last week”. So we could discuss it, and more specific” (HCP_GP2)* |

##### Subtheme 3B: What data to share and how to display it, and when

**Table 5.** Additional quotations stated while participants presented their own paper prototypes, grouped by code, about what data to share, how to share it and when to share it

| **Group** | **Code** | **Summary** | **Quotations** |
| --- | --- | --- | --- |
| **Participants with T1D** | How to share data | - Wish for HCPs to have access to patient-gathered data - Possibility of remote data-sharing - Preference to remotely conduct consultations when symptoms/ challenges happen | *“This is what I want and what I want my doctor to get access to, or nurse…. At least that my doctor to have easy access to my blood glucose. And I want him to get HbA1c and possible to see activity. Insulin at night if I bothered to” (T1D_P4)*  *“If I could say that now I’m struggling with something, and question if you [the HCP] could connect up and see the data…easier than booking an appointment” (T1D_P2)* |
|  | When to share data | - Prefer remote transfer of data - Prefer to be able to share data when it is needed, outside of consultations - Remote transfer of data outside of a consultation could allow HCPs to review it when they have time | *“Or I upload my data in my program and share with my nurse and then I get the question “Can you note this week what you put of insulin in the given period and then I get the data from you.” And then I don’t really have to meet up on this…and [I still] get specific answers on it, because then I feel I save a lot of time [for both myself and the nurse]. She can do it when she has the time for it…That is my dream situation” (T1D_P3)* |
| **Specialists** | What data to present | - Specific information patients should provide: insulin doses - Data should be accurate - Clinical changes depend on accurate data | *“They should know how much insulin they have taken per day, the last week at least or the last month and not give only an approximation because in case we are going to give them any advice on how they will change their medication we need to know what they actually have been taking” (Specialist2)* |
|  | Patients’ responsibilities | - Patient must collect data - Patient must be proactive/ prepare to present data - Sharing data can lead to better communication - Patient engagement can lead to better communication | *“You have the patient already before the consultation – trusting in her responsibility and her interest in doing better. Kind of her personal responsibility for it. As opposed to if a patient comes and has not gathered any data at all and the HbA1c was measured three months ago, and you don’t know how much insulin the patient uses and so on. So it will be a proactive attitude from the patient wants to better the communication and make the whole situation better. Both for patient and doctor” ( Specialist1)* |
|  | Concern: lack of communication | - Patients afraid to communicate – fear losing “privileges” - Communication is needed if provider is to offer guidance | *“In Denmark, there is this one study that has shown that after these rules [about how many instances of hypoglycaemias means a person is unfit to drive] were implemented then the patients with diabetes type 1 do not report their actual hypoglycaemias because they are afraid to lose their driving licence…it’s a problem of communication and openness and frankness between the patient and the doctor… I [would] not be able to give him any advice” (Specialist2)* |
| **Participants with T2D** | When to share data | - Preference to send data to GP prior to consultation - Pre-sharing data could be more efficient | “Data already be shown for the doctor before a consultation, so we have a baseline….To not waste time” (T2D_P1) |
|  | How to use a data-sharing system | - Use of the system changes based on diabetes duration - Assume HCPs do not want to store patient-gathered data - Information also about what data to present and how to present it | “I have set up these symbols, because you got to have something. When you get diagnosed with diabetes, [have to know] what you have to be aware of, and so [that information] is there about what you can do. Eventually get graphs and register schemas in and things. We have talked about the possibility of getting a compressed report that we could take to the doctor, since they don’t want any files” (T2D_P3) |
| **Exchange between participant with T2D and GP** | What to share | Participants clarifying assumptions about one another | Participant with T2D: *“Everything regarding medicines should be in doctor journal, so don’t think it’s important, it is self-measurements and blood glucose and physical activity and diet and what affects the blood glucose” (T2D_P1)*  GP: *“About medicine, we see in journal what we think you are taking, but we won’t know it is correct.” (GP1)* |
| **GPs** | Integrating data-sharing system into clinical practice | - Thought process about how to relate to data-sharing system - Present data and discussion according to patient’s duration of diabetes (time since diagnosis) - Alternative information sources when first diagnosed - Must consider time restrictions when discussing patient-gathered data | *“I drew it as if the paper prototype] was my computer at my office, because I think I would like this to be pretty easy in the start. Explain the high blood glucose, where are the limits, health consequences. The day you get diagnosed I think you blank out, and you don’t really know anything. So I would [present the data] very simply, and I would give them the website to Diabetes Association, where there is a lot information. And if [a patient] came to me and I have 20 minutes, [we would] look at blood glucose. I don’t have time to inform about [patients] about everything, so they have to look into it themselves. [We could] talk about physical activity, a bit on diet, what is good and not good. But don’t start with the apps or something at once – [do it] step by step. I would try to make it simple. [I would schedule] a new appointment pretty soon again – about 3 weeks. Then [we could] get into it a bit more and give it more thought” (GP1)* |
|  | Design of data-sharing system | - Needs to be simple - Synchronising data-exchange would be helpful - Ability to use data-sharing system on patients’ own computer | *“[I drew a] simple front page. [Maybe have] a synchronization with an app to phone and patient’s own computer. Same possibility for both. Simple front page, maybe a bit more thorough here. For example have an exercise diary with possibility to save data” (GP3)* |
|  | What data to share | Wellbeing | *“Wellbeing factor – yes useful…you get a greater understanding of the changes [of the other data types]” (GP2)* |
|  | How to share data | Patient must be present to share data | *“Without the patients, it is not useful. The patients should be there to use it for discussion and planning…knowing what’s going on” (GP2)* |
|  | When to share data | Do not want patients to share data prior to consultation | *“I DO NOT want [PGD] in advance [of the consultation]” (GP3)* |
